# *Salmonella enterica* serovar Typhi exposure elicits ex vivo cell-type-specific epigenetic changes in human gut cells

**DOI:** 10.1038/s41598-020-70492-2

**Published:** 2020-08-12

**Authors:** M. B. Sztein, A. C. Bafford, Rosângela Salerno-Goncalves

**Affiliations:** 1 Center for Vaccine Development and Global Health, Department of Pediatrics, University of Maryland School of Medicine, 685 West Baltimore Street, HSF 2, Room 480, Baltimore, MD 21201 USA; 2 Program in Oncology, University of Maryland Marlene and Stewart Greenebaum Comprehensive Cancer Center, Baltimore, MD 21201 USA; 3 Division of General and Oncologic Surgery, University of Maryland School of Medicine, Baltimore, MD 21201 USA

**Keywords:** Cell biology, Immunology, Microbiology

## Abstract

*Salmonella enterica* serovar Typhi (*S*. Typhi) causes substantial morbidity and mortality worldwide, particularly among young children. Humans develop an array of mucosal immune responses following *S*. Typhi infection. Whereas the cellular mechanisms involved in *S*. Typhi infection have been intensively studied, very little is known about the early chromatin modifications occurring in the human gut microenvironment that influence downstream immune responses. To address this gap in knowledge, cells isolated from human terminal ileum exposed ex vivo to the wild-type *S*. Typhi strain were stained with a 33-metal-labeled antibody panel for mass cytometry analyses of the early chromatin modifications modulated by *S*. Typhi. We measured the cellular levels of 6 classes of histone modifications, and 1 histone variant in 11 major cell subsets (*i.e*., B, CD3 + T, CD4 + T, CD8 + T, NK, TCR-γδ, Mucosal associated invariant (MAIT), and NKT cells as well as monocytes, macrophages, and epithelial cells). We found that arginine methylation might regulate the early-differentiation of effector-memory CD4+ T-cells following exposure to *S*. Typhi. We also found *S*. Typhi-induced post-translational modifications in histone methylation and acetylation associated with epithelial cells, NKT, MAIT, TCR-γδ, Monocytes, and CD8 + T-cells that are related to both gene activation and silencing.

## Introduction

*Salmonella enterica* serovar Typhi (*S*. Typhi) is an intracellular Gram-negative, rod-shaped bacteria that only infects humans by the fecal–oral route to cause typhoid fever^1–6^. *S*. Typhi causes substantial morbidity and mortality, particularly among younger children^7^. Following *S*. Typhi infection, humans develop an array of mucosal immune responses. Whereas the cellular mechanisms that determine *S*. Typhi infection have been intensively studied^2,3,8,9^, very little is known about the chromatin modifications occurring in the mucosal cells that influence downstream immune responses.

Epigenetic modifications, or marks including acetylation, methylation, phosphorylation, and ubiquitination, can alter gene activity, thereby regulating patterns of gene expression^10,11^. These processes are fundamental for the development and differentiation of distinct cell lineages in the body and can be modified by exogenous influences, such as environment and infectious diseases^11–14^. Bacterial-induced epigenetic changes may affect host cell function either by promoting host defense or by allowing pathogen persistence^15–17^. Thus, it is likely that exposure to bacterial organisms results in specific, long-lasting imprints on host cell responses to bacterial infection.

We hypothesized that gut colonization by *S*. Typhi causes epigenetic changes that are cell-type-specific. This hypothesis is based on our recent publications showing that *S*. Typhi strains with high degrees of homology, but small variations in gene expression, elicit differential host changes not only in the intestinal permeability and cytokine secretion but also in the phenotype and activation pathways of innate gut cells^8,9^. Here, we took advantage of the epigenetic landscape profiling (EpiTOF), a mass cytometry-based analytical platform that simultaneously measures epigenetic and immunological markers at the single-cell level^18,19^, to create an immune cell epigenetic atlas based on chromatin modification profiles. We used a 33-metal-labeled antibody panel and mucosal cells isolated from human terminal ileum tissues to generate novel information on early epigenetic modifications following ex vivo exposure to *S*. Typhi. We measured the cellular levels of 6 classes of histone modifications and one histone variant, in 11 major cell subsets (i.e., B, CD3+ T, CD4+ T, CD8 + T, NK, TCR-γδ, MAIT and NKT cells as well as monocytes, macrophages, and epithelial cells). By using high-dimensional analyzes of the data via t-Distributed Stochastic Neighbor Embedding (t-SNE)^20^ and self-organizing (FlowSOM)^21^ maps, we identified chromatin modifications between and within the different cell subtypes. We found that arginine methylation is one of the controllers of the early-differentiation of effector-memory CD4 + T-cells following exposure to *S*. Typhi. We also found *S*. Typhi-induced post-translational modifications in histone methylation and acetylation associated with epithelial cells, NKT, MAIT, TCR-γδ, Monocytes, and CD8 + T-cells that are related to both gene activation and silencing.

## Results

### Early epigenetic modifications following exposure to *S*. Typhi

**Figure 1:**
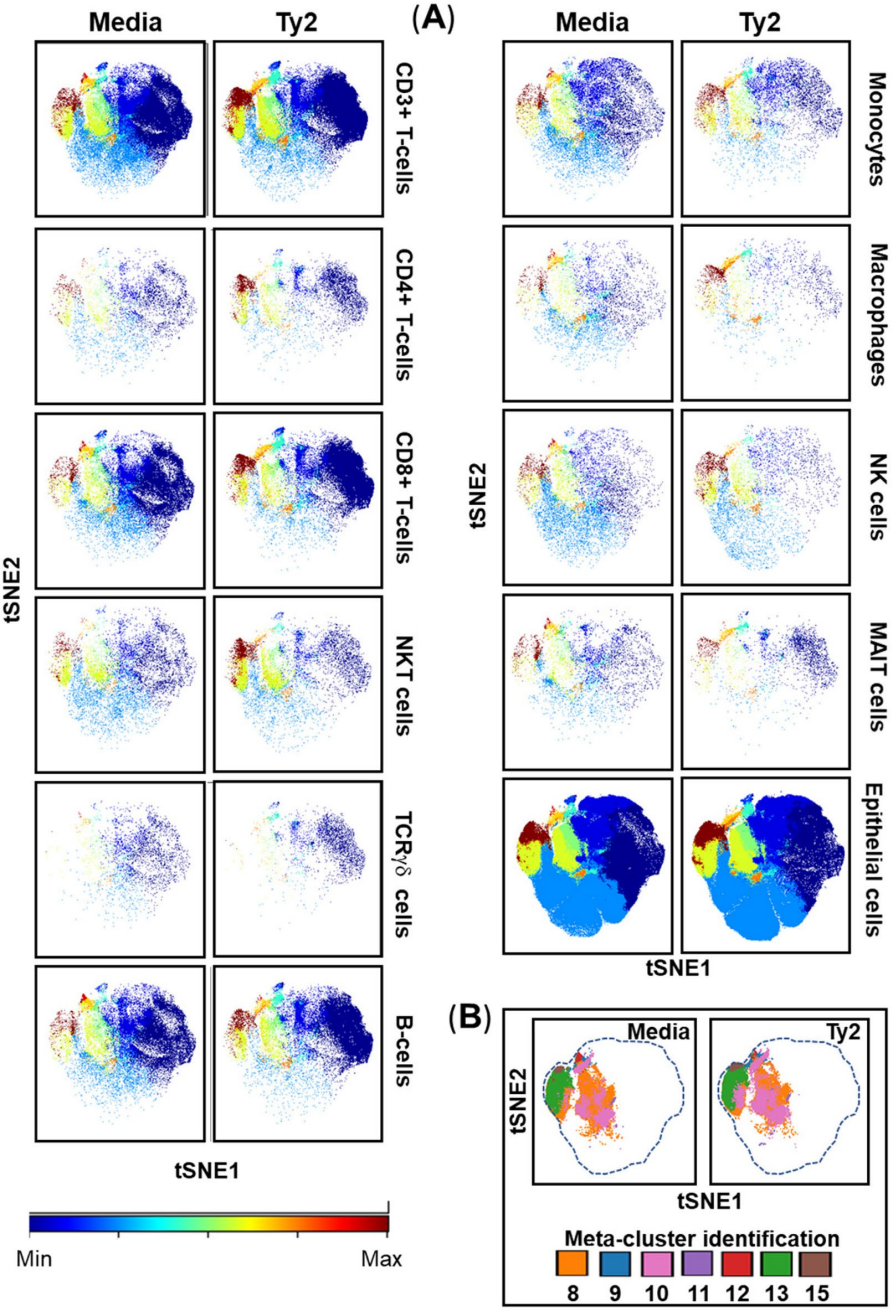
Unsupervised clustering of epigenetic changes between and within the different mucosal cell subsets after wild-type *S*. Typhi infections. Cells isolated cells from healthy terminal ileum surgical tissues were exposed to *S*. Typhi strain Ty2 (Ty2) at 1:100 MOI. Cells cultured with media only were used as controls (media). After 3 h of incubation, cells were stained using a panel of 33 metal-labeled Abs, and the chromatin modifications were analyzed at the single-cell level by mass cytometry. To visualize the clustering of the 11 cell subsets (*i.e*., B-, CD3+ T-, CD4+ T-, CD8 + T-, NK, TCR-γδ, Mucosal associated invariant (MAIT) and NKT cells, as well as monocytes, macrophages, and epithelial cells), tSNE-defined population distributions and clustering were colored by meta-cluster. (**A**) t-SNE maps of chromatin modifications. Settings to run the t-SNE algorithm were set-up in Cytobank. (**B**) Color-coded key showing the location of epigenetic marks meta-clusters. Data are representative of one out of three experiments with terminal ileum segments from 2 different donors, one replicate each.

**Figure 2:**
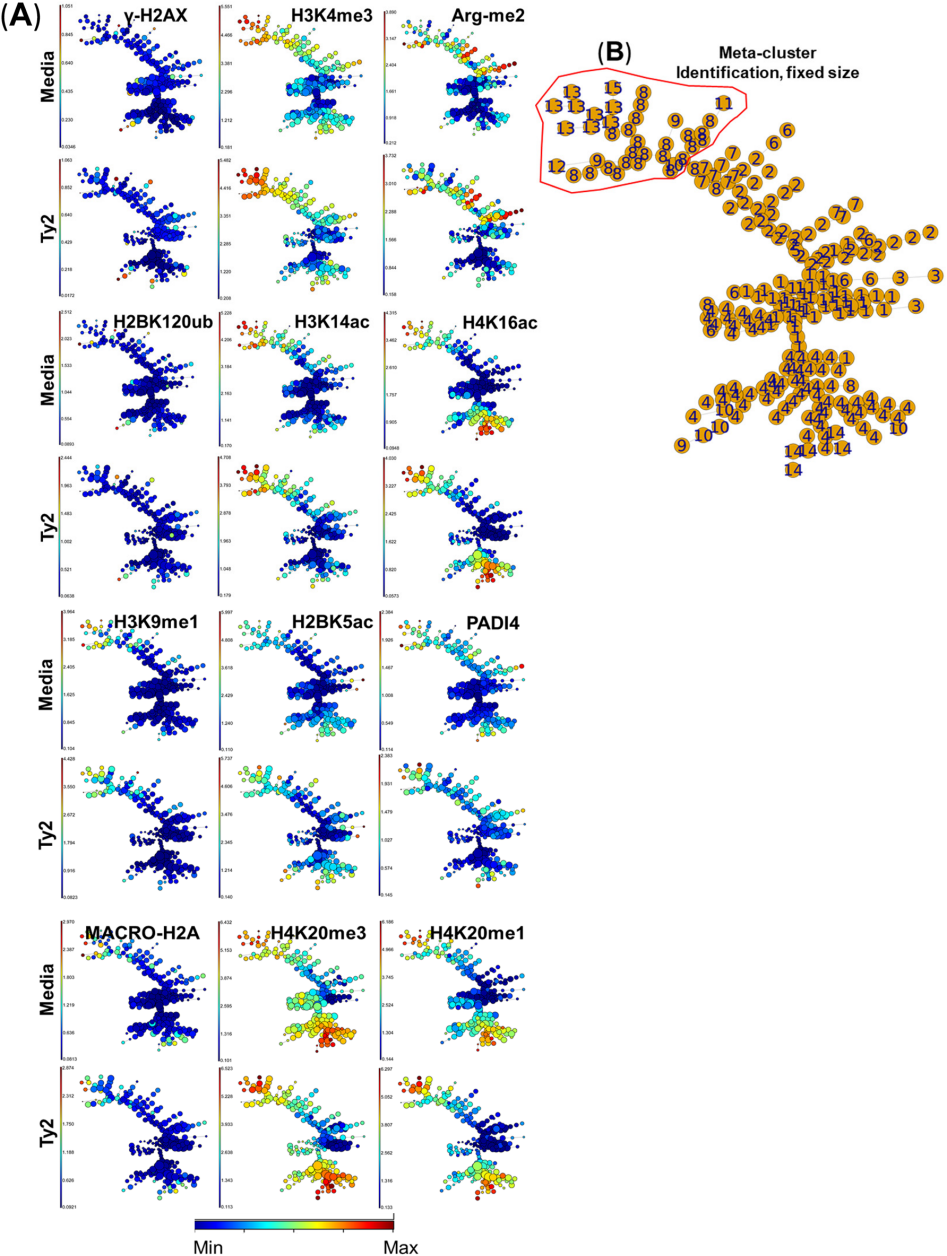
Unsupervised clustering of epigenetic changes associated with exposure to *S*. Typhi. Isolated cells from terminal ileum surgical tissues were exposed to *S*. Typhi strain Ty2 (Ty2) and cultured as described in Fig. 1. Cells cultured with media only were used as controls (media). (**A**) Using FlowSOM unsupervised clustering, 225 clusters were grouped into 15 meta-clusters of different sizes and organized based on their similarities in high-dimensional space. Settings to run the FlowSOM algorithm were set-up in Cytobank (see Materials and Methods). (**B**) Meta-cluster identification as observed in trees with nodes of a fixed size. Data are representative of one out of three experiments with terminal ileum segments from 2 different donors, one replicate each.

**Figure 3:**
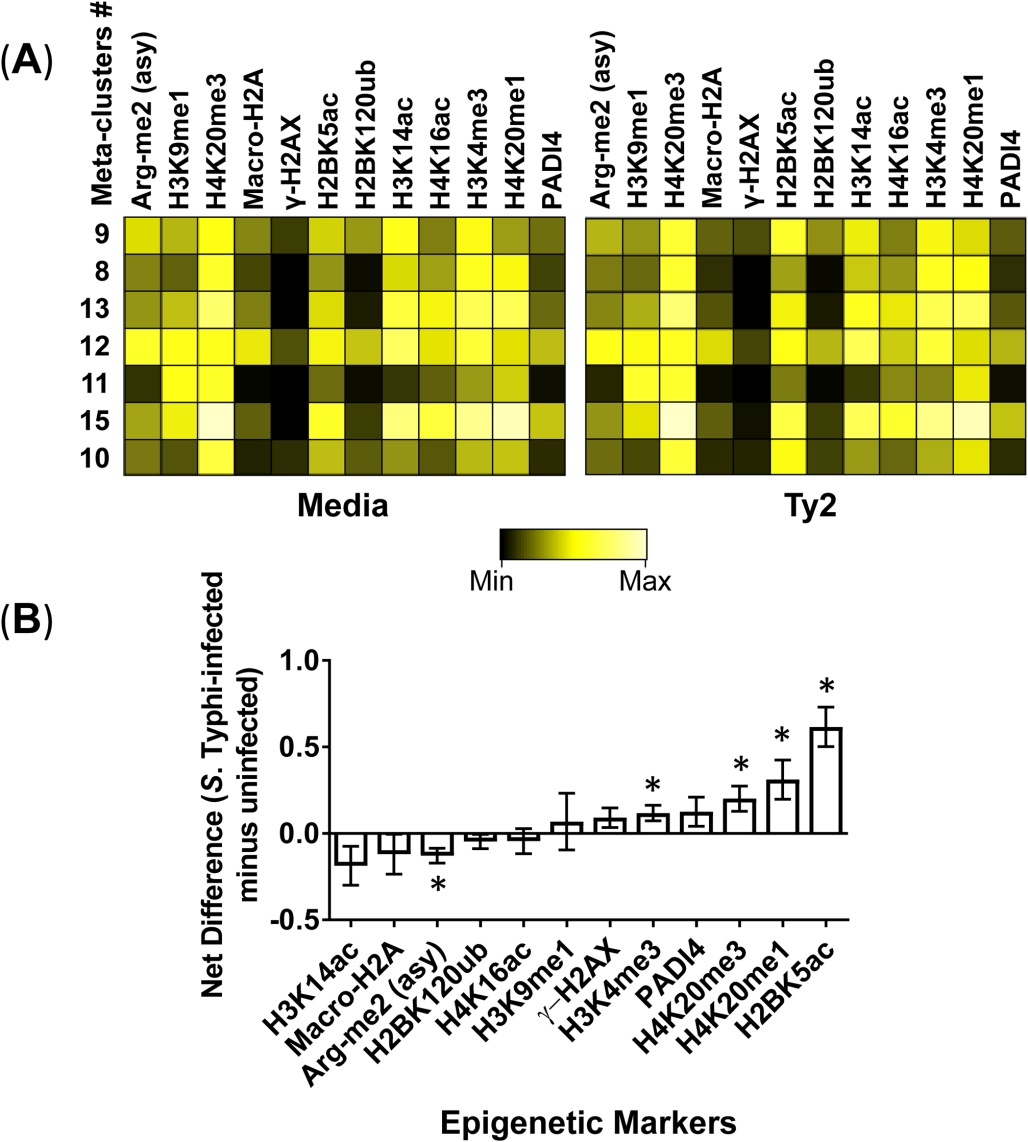
Chromatin changes elicited by *S*. Typhi infection. Cells isolated from healthy terminal ileum surgical tissues were exposed to *S*. Typhi strain Ty2 (Ty2) and cultured as described in Fig. 1. Cells cultured with media only were used as controls (media). (**A**) Heatmap of unsupervised hierarchical clustering of the 13 markers specific to 6 histone modifications and one histone variant. Heatmap colors represent the relative difference in the transformed ratio of the median detection of the markers, from low (black) to high (bright yellow). Each heatmap uses the same minimum and maximum gradient scale values. These values are calculated automatically in Cytobank and are based on the transformed ratio of the minimum value in the heatmap compared to each data point within the heatmap. The seven meta-clusters showing the greatest changes in the FlowSOM unsupervised clustering were analyzed. (**B**) The transformed ratio of the median of specific chromatin markers associated with the seven meta-clusters was assembled, and the differences in their detections between uninfected (media) and *S*. Typhi (Ty2) cultures were evaluated. *P*-values between uninfected and *S*. Typhi-infected cultures are from paired Student’s t-tests. *P* values < 0.05 were considered significant. Bar graphs represent the mean of the pooled data. The whiskers delineate the standard errors. Data are representative of one out of three experiments with terminal ileum segments from 2 different donors, one replicate each.

**Figure 4:**
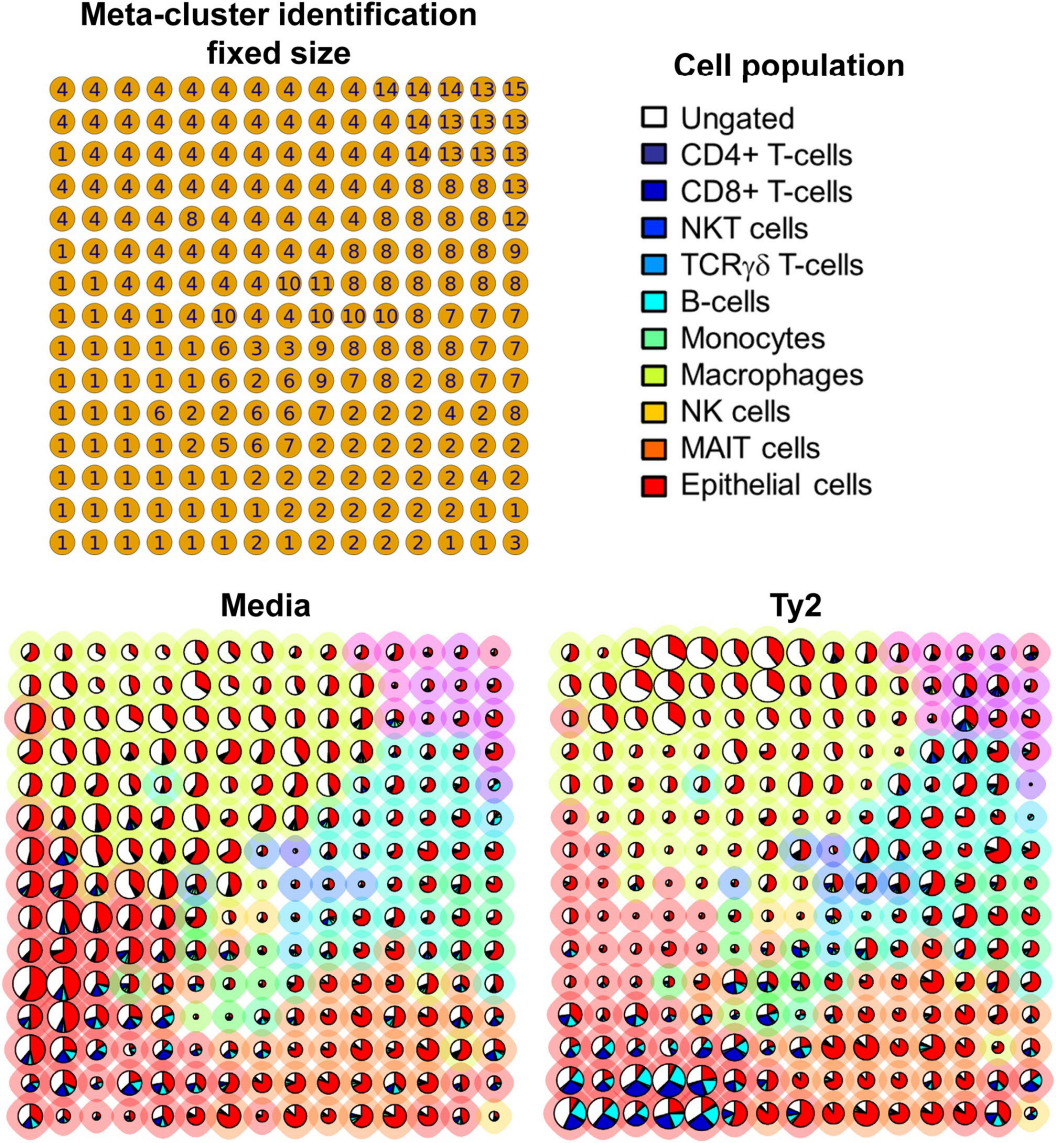
Meta-cluster maps of mucosal cell subset abundances. Cells isolated from healthy terminal ileum surgical tissues were exposed to *S*. Typhi strain Ty2 (Ty2) and cultured as described in Fig. 1. Cells cultured with media only were used as controls (media). We used FlowSOM’s built-in pie charts to assess how the FlowSOM meta-clustered data of the 13 markers specific to 6 histone modifications and one histone variant relate to the abundance of the different mucosal cell subsets. The background halo of each pie indicates its meta-cluster assignment, and its associated identification number is shown in the *top-left* grid. Each pie slice indicates the proportion of the selected manually gated cell population (*top-right* legend), and the pie size corresponds to the average representation of the population. These data are representative of one out of three experiments with terminal ileum segments from 2 different donors, one replicate each.

To functionally evaluate bacterial-induced epigenetic changes on human mucosal cells, we isolated cells from terminal ileum surgical tissues collected during clinical care of adults, that were subsequently exposed ex vivo to *S*. Typhi strain Ty2. Cells cultured in media only were used as negative controls. After 3 h of incubation, cells were labeled using a panel of 33 metal-labeled Abs (Supplementary Table 1) to identify chromatin modifications at the single-cell level in the various cell populations. We measured the cellular levels of 6 classes of histone modifications and one histone variant (Supplementary Table 2) in 11 major cell subsets (i.e., B, CD3 + T, CD4 + T, CD8 + T, NK, TCR-γδ, MAIT and NKT cells, as well as monocytes, macrophages, and epithelial cells). Of importance, 8–10 million individual cells were analyzed for each volunteer in each experiment to assure that sufficient numbers of events are analyzed for each subset*.* The high-dimensional and single-cell nature of these datasets allowed the creation of an immune cell epigenetic atlas based on cell lineage markers and chromatin modification profiles. When comparing t-SNE maps of uninfected (media) with those of *S*. Typhi-infected (Ty2) cultures, we observed that chromatin changes occurred in all manually gated cell subsets (Fig. 1A). Differences were more pronounced in meta-clusters 8, 9, 10, 11, 12, 13, and 15 compared with the other 8 remaining meta-clusters (1, 2, 3, 4, 5, 6, 7, and 14) (Fig. 1B). To confirm the presence, relative abundance, and changes in these meta-clusters, we next performed FlowSOM unsupervised meta-clustering tree analyses of chromatin modifications, which hierarchically ordered the meta-clusters based on their similarity and detection level. Using this approach, we found that similar to t-SNE maps, meta-clusters 9, 8, 13, 12, 11, 15, and 10 were associated with chromatin changes observed following Ty2 infection (Fig. 2A,B). Comparative analysis between the results of media and Ty2 cultures using heatmaps allowed us to find significant increases in the proportions of H3K4me3, H4K20me3, H4K20me1, and H2BK5ac marks contained in the 9, 8, 13, 12, 11, 15, and 10 meta-clusters (Fig. 3A,B). We also found significant decreases in the Arg-me2 (asy) mark contained in the same meta-clusters (Fig. 3A,B). Next, we used a pie grid to visualize the effects of Ty2 infection using FlowSOM analyses. Of note, while the detection levels of chromatin marks changed in immune cells contained in 9, 8, 13, 12, 11, 15, and 10 meta-clusters, major changes in the immune cell population size also occurred in meta-cluster 1, which did not show increases in the detection levels of chromatin marks used in this study (Fig. 4). We also observed that not all populations having chromatin changes were represented in the cell subsets being evaluated (*e.g*., ungated population, meta-cluster 4) (Fig. 4). Thus, the evaluation of supplementary epigenetic markers is necessary to identify specific epigenetic marks, such as the ones observed meta-cluster 1. Supplementary cell lineage markers are also needed to identify additional cell subsets, such as the ones observed meta-cluster 4. Finally, our data suggest concomitant changes in histone methylation and acetylation after Ty2 infection.

### Multifunctional pattern of the epigenetic changes in mucosal cells

**Figure 5:**
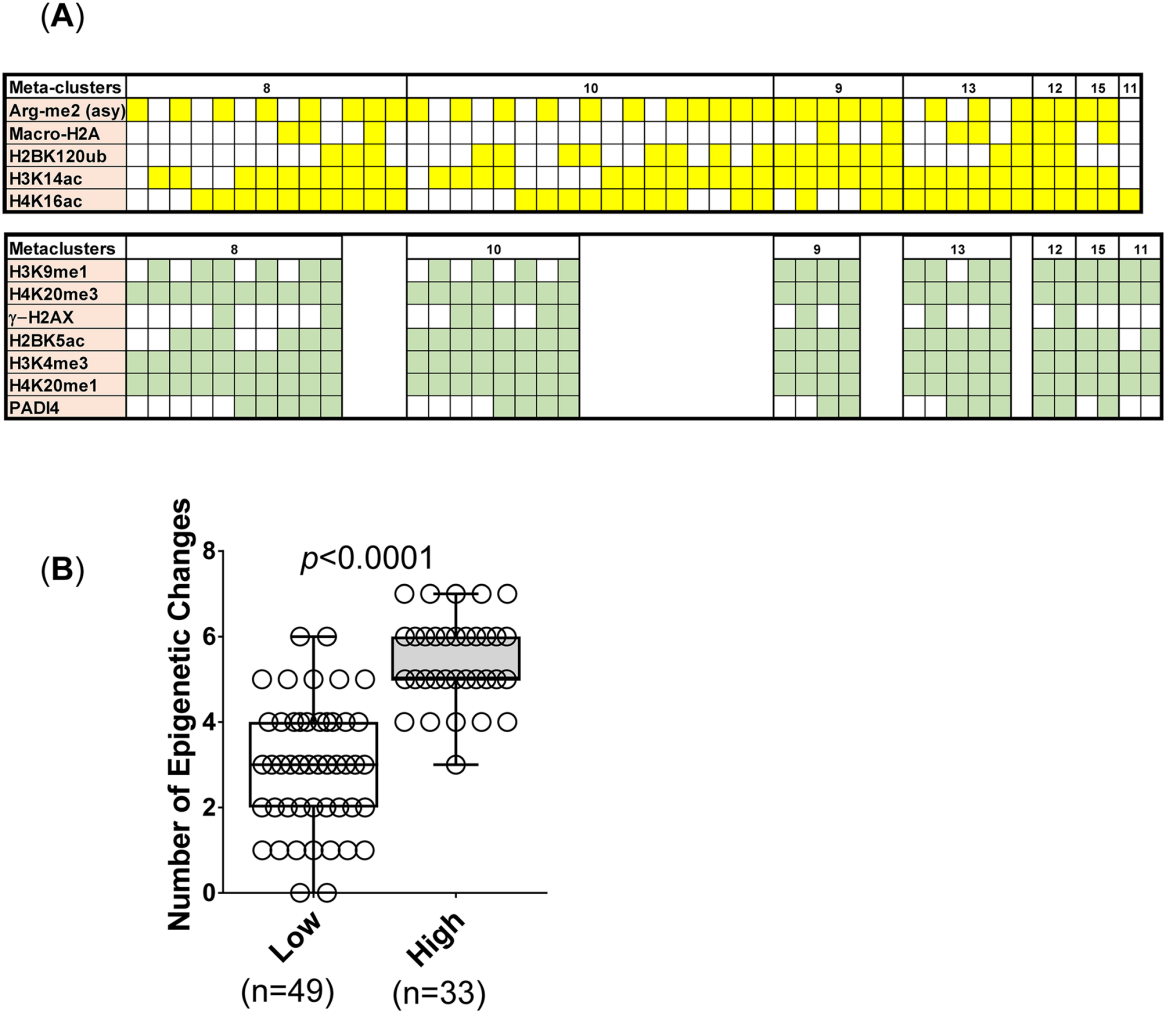
Multifunctional patterns of the epigenetic changes in mucosal cells induced by *S*. Typhi. Cells isolated cells from healthy terminal ileum surgical tissues were exposed to *S*. Typhi strain Ty2 (Ty2) and cultured as described in Fig. 1. Cells cultured with media only were used as controls (media). (**A**) Cells were grouped based on the detection levels of the chromatin markers (*i.e., top row,* decreases [Arg-me2 (asy), Macro-H2A, H2BK120ub, H3K14ac, and H4K16ac]; and *bottom row,* increases [H3K9me1, H4K20me3, γ-H2AX, H2BK5ac, H3K4me3, H4K20me1, and PADI4]). FCOM analyses were used to evaluate the changes based on the 32 possible combinations for the 5 epigenetic markers in the group showing decreases, and the changes based on the 128 possible combinations for the 7 markers in the group showing increases. Data show all the changes that occurred after Ty2 exposure. (**B**) Comparison between the number of epigenetic changes between the group showing decreases (low) and the group showing increases (high) in the chromatin marks. *P* values < 0.05 were considered significant. Bar graphs contain all combinations observed in 8, 10, 9, 13, 12, 15 and 11 meta-clusters in each of the 2 groups. Bar graphs extend from the 25th to 75th percentiles; the line in the middle represents the median of the pooled data. The whiskers delineate the smallest to the largest value. These data are representative of one out of three experiments with terminal ileum segments from 2 different donors, one replicate each.

Since the above data suggest concurrent (*i.e.,* concomitantly positive for two or more marks) detection of multiple chromatin marks, we next studied the multifunctional pattern of the chromatin marks in the mucosal cells induced by Ty2. We divided the chromatin marks into two groups based on the results in Fig. 3: (1) marks with increased detection (either showing significant increases or trends to show increases; *i.e*., H3K9me1, γ-H2AX, H3K4me3, PADI4, H4K20me3, H4K20me1, and H2BK5ac), and (2) marks with decreased detection (either showing significant decreases or trends to show decreases; *i.e*., H3K14ac, Macro-H2A, Arg-me2 (asy), H2BK120ub, H4K16ac) (Fig. 3B). Mass cytometry data from live gated cells were analyzed using the FCOM function of Winlist software to determine the proportion of chromatin changes in the 32 possible combinations for the 5 epigenetic markers in the group showing decreases, and in the 128 possible combinations for the 7 markers in the group showing increases. Chromatin changes were considered evaluable if the number of positive events was more than 6 for immune cells, and more than 30 in the gate defined for epithelial cells (~ 80% of the cells in the preparations). In the group with increased detection of chromatin marks, we found changes in 11 out of the 128 possible combinations. The pattern of these changes was tilted towards a concomitant detection of multiple chromatin marks, particularly the ones associated with methylation of H3 at lysine 4 (H3K4me3) and H4 at lysine 20 (H4K20me3 and H4K20me1) (Fig. 5A). In the group with decreased detection of chromatin marks, we found changes in 17 out of the 32 possible combinations. The pattern of these changes was towards the detection of chromatin marks associated with arginine methylation (Arg-me2(asy)), and H3 and H4 acetylation (H3K14ac and H4K16ac) (Fig. 5A). When comparing the number of epigenetic changes between the two groups, we observed a significant increase in the number of live cells with multiple chromatin marks in the group showing increased detection as compared to the group showing decreased detection (Fig. 5B) (*p* < 0.0001). Therefore, it is reasonable to hypothesize that the changes in these chromatin marks may occur simultaneously and be present in multiple immune cell subsets.

### Chromatin profiles are cell-type specific

**Figure 6:**
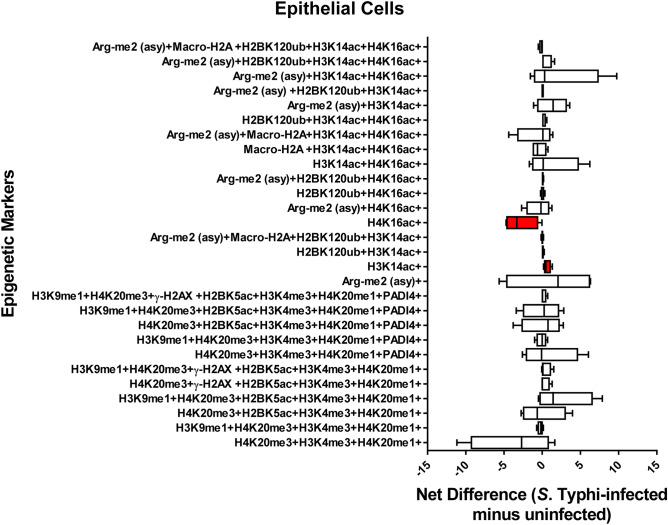
Chromatin profiles of the epigenetic changes in epithelial cells induced by *S*. Typhi. Cells isolated cells from healthy terminal ileum surgical tissues were exposed to *S*. Typhi strain Ty2 (Ty2) and cultured as described in Fig. 1. Cells cultured with media only were used as controls (media). FCOM data of the 28 combinations within the acceptability criteria for changes of the chromatin marks are shown. Bars represent the net difference (*S*. Typhi-infected minus uninfected cultures). Bar graphs extend from the 25th to 75th percentiles; the line in the middle represents the median of the pooled data. The whiskers delineate the smallest to the largest value. Data are representative of three experiments with terminal ileum segments from 4 different donors, one replicate each. *P* values < 0.05 were considered significant (red-colored boxes).

**Figure 7:**
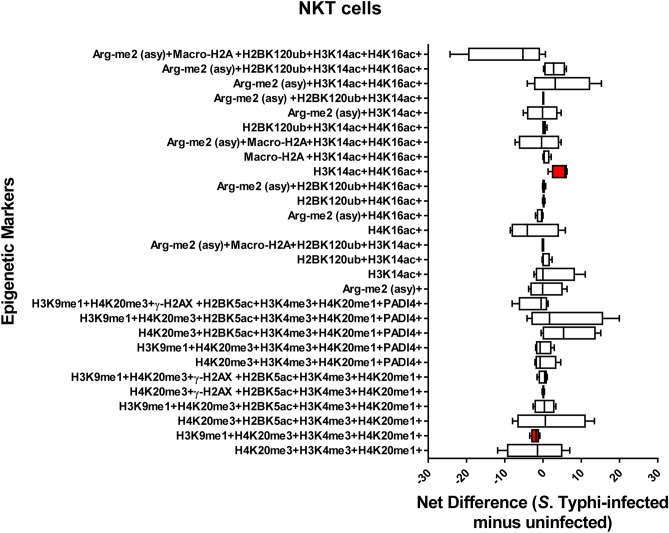
Chromatin profiles of the epigenetic changes in NKT cells induced by *S*. Typhi. Cells isolated cells from healthy terminal ileum surgical tissues were exposed to *S*. Typhi strain Ty2 (Ty2) and cultured as described in Fig. 1. Cells cultured with media only were used as controls (media). FCOM data of the 28 combinations within the acceptability criteria for changes of the chromatin marks are shown. Bars represent the net difference (*S*. Typhi-infected minus uninfected cultures). Bar graphs extend from the 25th to 75th percentiles; the line in the middle represents the median of the pooled data. The whiskers delineate the smallest to the largest value. Data are representative of three experiments with terminal ileum segments from 4 different donors, one replicate each. *P* values < 0.05 were considered significant (red-colored boxes).

**Figure 8:**
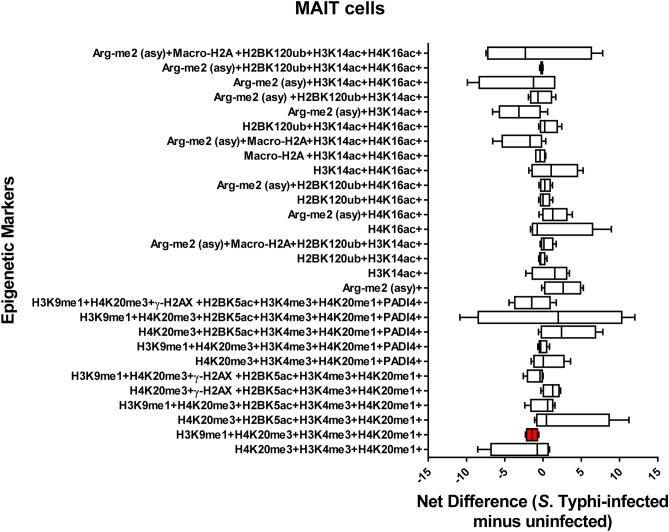
Chromatin profiles of the epigenetic changes in MAIT cells induced by *S*. Typhi. Cells isolated cells from healthy terminal ileum surgical tissues were exposed to *S*. Typhi strain Ty2 (Ty2) and cultured as described in Fig. 1. Cells cultured with media only were used as controls (media). FCOM data of the 28 combinations within the acceptability criteria for changes of the chromatin marks are shown. Bars represent the net difference (*S*. Typhi-infected minus uninfected cultures). Bar graphs extend from the 25th to 75th percentiles; the line in the middle represents the median of the pooled data. The whiskers delineate the smallest to the largest value. Data are representative of three experiments with terminal ileum segments from 4 different donors, one replicate each. *P* values < 0.05 were considered significant (red-colored box).

**Figure 9:**
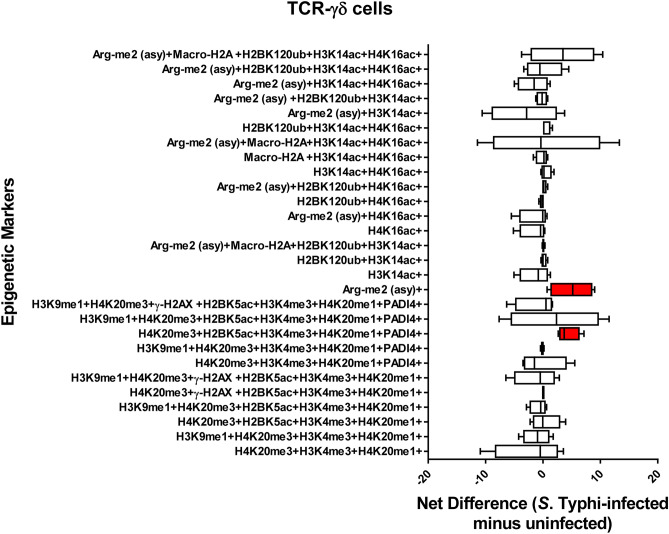
Chromatin profiles of the epigenetic changes in TCR-γδ cells induced by *S*. Typhi. Cells isolated cells from healthy terminal ileum surgical tissues were exposed to *S*. Typhi strain Ty2 (Ty2) and cultured as described in Fig. 1. Cells cultured with media only were used as controls (media). FCOM data of the 28 combinations within the acceptability criteria for changes of the chromatin marks are shown. Bars represent the net difference (*S*. Typhi-infected minus uninfected cultures). Bar graphs extend from the 25th to 75th percentiles; the line in the middle represents the median of the pooled data. The whiskers delineate the smallest to the largest value. Data are representative of three experiments with terminal ileum segments from 4 different donors, one replicate each. *P* values < 0.05 were considered significant (red-colored boxes).

**Figure 10:**
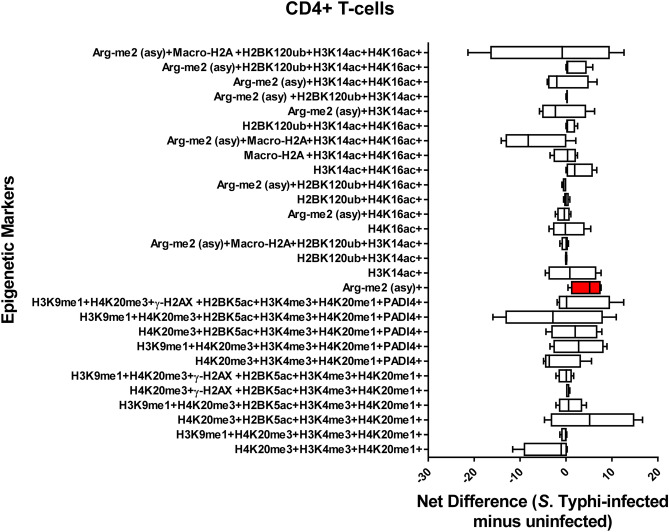
Chromatin profiles of the epigenetic changes CD4 + T-cells induced by *S*. Typhi. Cells isolated cells from healthy terminal ileum surgical tissues were exposed to *S*. Typhi strain Ty2 (Ty2) and cultured as described in Fig. 1. Cells cultured with media only were used as controls (media). FCOM data of the 28 combinations within the acceptability criteria for changes of the chromatin marks are shown. Bars represent the net difference (*S*. Typhi-infected minus uninfected cultures). Bar graphs extend from the 25th to 75th percentiles; the line in the middle represents the median of the pooled data. The whiskers delineate the smallest to the largest value. Data are representative of three experiments with terminal ileum segments from 4 different donors, one replicate each. *P* values < 0.05 were considered significant (red-colored box).

We next investigated whether there were relationships between different cell populations and the detection chromatin marks after exposure to Ty2. To this end, we investigated the 28 possible FCOM phenotypes (11 and 17 in the group with increased and decreased detection of chromatin marks respectively) on 11 major cell subsets (*i.e*., B, CD3 + T, CD4 + T, CD8 + T, NK, TCR-γδ, MAIT cells, NKT cells, monocytes, macrophages, and epithelial cells). Chromatin changes specific to exposure to *S*. Typhi were calculated as the differential in the number of positive events between experimental (Ty2) and negative control (media) cultures. We found that Ty2 infection modified the detection of chromatin marks in the function of the individual cell subsets. Epithelial cells showed a significantly increased detection of histone acetylation H3K14ac, but significantly decreased detection of histone acetylation H4K16ac (Fig. 6). NKT cells also showed changes in the detection of histone acetylation (H3K14ac, H4K16ac) (Fig. 7). Immune cells, such as NKT (Fig. 7), MAIT (Fig. 8), TCR-γδ (Fig. 9), Monocytes (Supplementary Fig. 2), and CD8 + T-cells (Supplementary Fig. 3) showed changes towards a concomitant detection of multiple chromatin marks, particularly the ones associated with methylation of H3 at lysine 4 (H3K4me3) and H4 at lysine 20 (H4K20me3 and H4K20me1). Like TCR-γδ cells (Fig. 9), CD4 + T-cells (Fig. 10) showed significantly increased detection of the arginine methylation ((Arg-me2 (asy)) mark after Ty2 infection. Trends of increased detection of Arg-me2 (asy) marks were also observed in MAIT and CD3 + T-cell populations (Fig. 8 & Supplementary Fig. 4). Of note, macrophages (Supplemental Fig. 5), NK (Supplemental Fig. 6), B-cells (Supplemental Fig. 7), and CD3 + T-cells (Supplementary Fig. 4) showed no significant changes in the detection on any of the 28 possible FCOM phenotypes after Ty2 infection. Finally, as shown in Supplemental Fig. 8, there were not significant bulk histone changes in response to *S*. Typhi infection. The mean of H3 histone distribution was 1.1 ± 0.43 (mean ± 2SD) (ranging from 0.7 to 1.5), with non-significant changes in any of the 11 different cell subsets evaluated. In summary, our data support the hypothesis that Ty2 infection regulates concurrent changes of functionally related chromatin marks that depends on the individual cell types and subsets.

### Hierarchical clustering of the cell subsets using Principal Component Analysis (PCA)

To confirm and extend the FCOM results, PCA was performed on the same data set. FCOM data from all replicates were merged for combined analysis, increasing the statistical power, and creating a matrix of 28 possible FCOM phenotypes in 11 cell subsets (308 points). PCA of 308 data points revealed that the first 3 principal components (PC1, PC2, and PC3) accounted for 73% of the total variance (Supplemental Fig. 9A), and cultures exposed to Ty2 were distinguished from media (control) mainly based on PC2 values rather than by PC1 (Supplemental Fig. 9B) and PC3 values (data not shown). Analysis of PC2 loadings indicates that the main variances occurred in cells showing: (1) Arg-me2(asy)  + , (2) Arg-me2(asy) + Macro-H2A + H2BK120ub + H3K14ac + H4K16ac + , (3) H3K9me1 + H4K20me3 + H2BK5ac + H3K4me3 + H4K20me1 + PADI4 + , and (4) H3K9me1 + H4K20me3 + γ-H2AX + H2BK5ac + H3K4me3 + H4K20me1 + PADI4 + phenotypes (Supplemental Fig. 9C). Cells showing (1) H3K14ac + , (2) Arg-me2(asy) + Macro-H2A + H3K14ac + H4K16ac + , (3) H4K20me3 + H3K4me3 + H4K20me1 + , (4) H3K9me1 + H4K20me3 + H2BK5ac + H3K4me3 + H4K20me1 + , and (5) Arg-me2 (asy) + H3K14ac + H4K16ac + contributed to a lesser extent (Supplemental Fig. 9C). We next performed cell hierarchical clustering analyses (Supplemental Fig. 10). It is interesting to note the arrangement of these phenotypes suggests a connection between Arg-me2(asy) and H4K20me3, H3K4me3, H4K20me1, which were then connected to PADI4. Except for epithelial cells (Supplemental Fig. 11), and at different levels, these marks also displayed high variances between media control and Ty2 infection when analyzing individual populations such as NKT cells (Supplemental Fig. 12), MAIT cells (Supplemental Fig. 13), TCRγδ cells (Supplemental Fig. 14), and CD4 + T-cells (Supplemental Fig. 15). Together, these results indicate that following *S*. Typhi exposure, cells undergo multiple coordinated epigenetic modifications, which are cell subset specific, although they share similarities among them.

**Figure 11:**
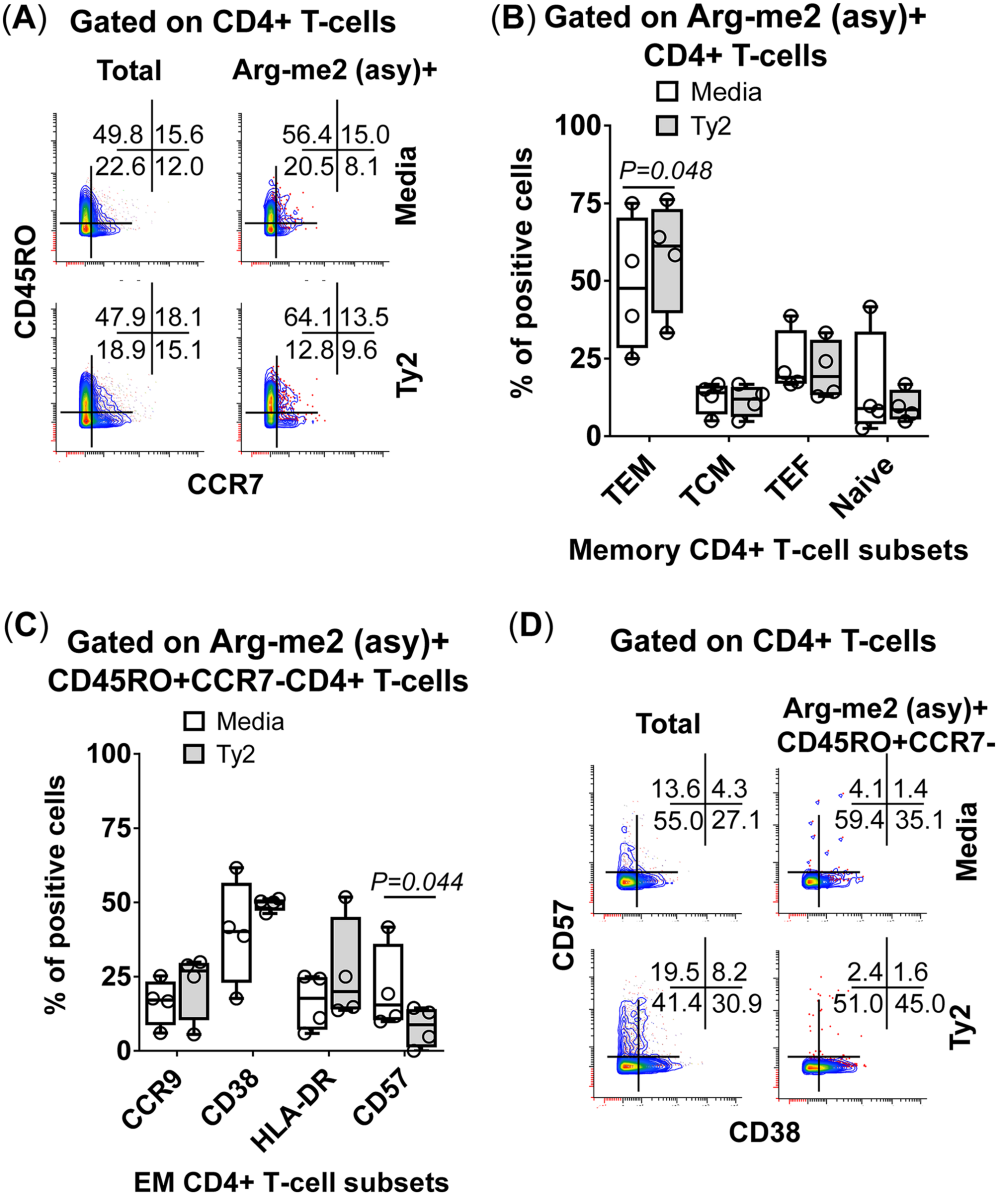
Phenotype of Arg-me2 (asy) + CD4+ T-cells induced by *S*. Typhi. Detection of Arg-me2 (asy) + in CD4+  T-cells and association with functionality as measured by mass cytometric analyses of **(A)** memory markers: effector memory (TEM, CD45RO + CCR7-), central memory (TCM, CD45RO + CCR7 +), effector (TEF, CD45RO-CCR7-), and naïve (CD45RO-CCR7 +) populations. **(B)** Bars represent the mean expression of the TEM, TCM, TEF and naïve in *S*. Typhi-infected (gray bars) and uninfected (open bars) cultures in the 4 volunteers. (**C**) Expression of homing (CCR9 +), activation (CD38 + and HLA-DR +), and maturation (CD57 +) markers in *S.* Typhi-infected (gray bars) and uninfected (open bars) cultures in the 4 volunteers. (**D**) CD57 and CD38 expression on Arg-me2 (asy) + CD45RO + CCR7-CD4 + T-cells in a representative volunteer. Bar graphs (**B, C**) extend from the 25th to 75th percentiles; the line in the middle represents the median of the pooled data. The whiskers delineate the smallest to the largest value. Data are representative of three experiments with terminal ileum segments from 4 different donors, one replicate each. *P* values < 0.05 were considered significant.

### Analyses of phenotypic changes on Arg-me2 (asy) + CD4 + T-cells

To further investigate the relationship between the increased frequency of Arg-me2 (asy) + CD4 + T-cells and their functionality, we measured memory (CD45RO)^22–24^, homing (CCR7 and CCR9)^25^, activation (CD38 and HLA-DR)^25^, and maturation (CD57)^25–27^ markers on Arg-me2 (asy) + CD4 +  T-cells. Different memory T cell subsets were divided based on their expression of CD45RO and CCR7 markers into central memory T (TCM) cells (CD45RO + CCR7 +), effector memory T (TEM) cells (CD45RO+ CCR7-), and effector T (TEF) cells (CD45RO-CCR7-). We found that Arg-me2 (asy) + CD4 + TEM T-cells were enriched in cultures following exposure to Ty2 (Fig. 11A & B). We also used the expression of CCR9, CD38, and CD57 to characterize Arg-me2 (asy) + TEM CD4 + T-cells further. We observed a significant decrease in Arg-me2 (asy) + TEM CD4 + T-cells expressing CD57 markers after Ty2 exposure (Fig. 11C & D). Of note, as shown in Fig. 11D, the frequency of Arg-me2 (asy) + TEM CD4 + T-cells expressing CD57 markers was consistently lower than those observed in total CD4 + T cells, suggesting that Arg-me2 (asy) chromatin perturbations occur on CD4 + T-cells with phenotypic features of immature cells (CD57-). Interestingly, it is likely that a sizable proportion of these CD4 + T-cells were activated (CD38 +) (Figs. 11C & D). Thus, *S*. Typhi exposure triggered arginine methylation (Arg-me2 (asy)) in multiple CD4 + T-cell subsets with diverse characteristics associated with memory, maturity, and activation.

## Discussion

Post-translational modifications include acetylation, methylation, phosphorylation, and ubiquitination. These modifications are known to dictate chromatin structure and function, which leads to changes in cell behavior and, consequently, tissue development^28^. In many cases, single and/or multiple histone modifications occur in the same position affecting the level of genomic control^29^. Upon recognition of microbial components or its products through host pattern recognition receptors (PRRs), a cascade of intracellular transcription factors is activated, which ultimately regulate genes involved in host responses^11^. Alterations in chromatin marks negatively or positively regulate these pathways to suppress or activate mucosal cells. The data herein supports the assumption that *S*. Typhi infection results in epigenetic modifications that are cell type and subset specific. These include post-translational modifications (*e.g.,* methylation of H3 at lysine 4 (H3K4me3) and H4 at lysine 20 (H4K20me3 and H4K20me1), acetylation (H3K14ac and H3K16ac) and arginine methylation (Arg-me2(asy)) that are poised to affect cell differentiation. Specific epigenetic modifications, including histone modifications, may accompany T-cell differentiation and allow the expression of genes associated with that lineage^30^. This contention is also supported by previous studies showing that H4K20 monomethylation (H4K20me1) regulates both H4K16Ac and H4K20me3^31,32^. H4K20me1 not only is required for H4K16 acetylation, but also the di- and trimethylation of H4K20, and the local conversion of H4K20me1 to H4K20me3^31,32^. It is well established that in mammals, the states of the different H4K20 methylation states are established through specific enzymes^32^. In this regard, additional kinetic studies are needed to establish temporal patterns and define the role of these modifications and associated catalyzing enzymes in chromatin signaling and subsequent development of host immune responses.

We also observed increased detection of the arginine methylation (Arg-me2 (asy)) mark in TEM CD4+ T-cells following *S*. Typhi infection. These results are in agreement with previous data showing that naïve B- and T-cells are less sensitive to perturbations of arginine methylation than memory cells^33,34^. Arginine methylation also controls the strength of cell signaling via γ chain (γc)-family cytokines, including interleukin-2 (IL-2), IL-4, IL-7, IL-9, IL-15, and IL-21^35^, that are essential for T-cell development and function^36^. Also, we established that arginine methylation involved TEM CD4+ T cells frequently lacking the expression of CD57, a molecule that is associated with cell maturation^26^. Previous work has suggested that CD57 negative T-cells cells are often polyclonal^37^ and able to recognize a wide range of antigens. Thus, it is reasonable to assume that early-differentiated CD57 negative TEM CD4 + T-cells in the human gut readily increase the detection of Arg-me2 (asy) following *S*. Typhi stimulation, likely leading to the fast production of multiple cytokines.

Cellular metabolites/cofactors such as S-adenosyl methionine (SAM), Fe2 +/Fe3 + , and α-ketoglutarate are known to modulate Histone methylation/demethylation^38^. Thus, it is reasonable to hypothesize that metabolites elicited by *S*. Typhi infection influence the activity of enzymes participating in histone methylation. This hypothesis is based on a recent study showing that the microbiota, in combination with dietary substrates, produce metabolites that affect histone methylation in multiple tissues of the host^39^. In particular, short-chain fatty acids (SCFAs), major products of gut microbiota fermentation, are modulated by the host epigenome through DNA methylation and histone modifications^29,39^. *S*. Typhi also uses transcriptional changes, such as the rearrangement of the cellular machinery of the host cytoskeleton to promote bacterial invasion and production of cytokines, which are likely to help them escape intestinal immune defense responses^8,9,40^. Indeed, by using a wild-type *S*. Typhi challenge model, our group had shown that differences related to redox potential and ion homeostasis in the gut microbiota might impact clinical outcomes following exposure to wild-type *S*. Typhi infection^41^. Further studies are needed to identify the specific molecules and modes of action by which *S*. Typhi directly or indirectly influences the epigenome and the consequences of these regulatory processes. Previously, our group has shown that *S*. Typhi triggers intestinal cell responses that are distinct from the ones triggered by their counterparts in the periphery^42^. We have also shown that multiple cell subsets are involved in these host responses, including TEM CD4 + and CD8 + subsets, as well as tissue-resident memory T-cells^42,43^. Consequently, understanding the action of epigenetic regulators, which determine defined transcription patterns of the genes in different cell types and subsets, will be essential in characterizing the pathways associated with protection from infection through the development of robust and effective host immune responses.

Finally, it is not possible to exclude that some of the observations and differences highlighted in this study were due, to some extent, to the fact that these studies were performed with cells isolated from healthy tissues obtained from individuals with inflammatory bowel disease. Cells isolated from patients with inflammatory bowel disease might respond to external stimuli differently from healthy individuals^44^. Thus, future investigations using cells from healthy human terminal ileum are needed to confirm and extend the role of these epigenetic mark changes in the context of *S*. Typhi infection.

In summary, to our knowledge, this study provided the first proof of principle that early epigenetic changes in mucosal cells isolated from the human terminal ileum can be directly linked to exposure to a major gastrointestinal bacterial pathogen. This study also showed that these changes are cell type and subset specific and related to histone methylation, acetylation, and arginine methylation that are associated with both gene activation and silencing.

## Methods

### Human tissues

Non-diseased terminal ileal tissue from four patients with inflammatory bowel disease (IBD), aged 24–72 years, was collected at the time of routine surgery and used to isolate epithelial and immune cells. The tissue was determined to be normal based on a gross inspection by the surgeon. Specimens were de-identified and replaced with random numbers and letters. The only data retained from these specimens were the approximate date of collection (month/year), gender, and age. A protocol describing the collection and use of these specimens was submitted to the University of Maryland IRB, and a study exemption (*i.e*., not human research determination) was approved (#HP-00077485). All tissues were processed within 1 h of collection. Cells were isolated as described below and cryopreserved in cell freezing medium (*i.e.,* 50% Fetal Bovine Serum and 10% dimethyl sulfoxide (DMSO) in DMEM) and storage in liquid N_2_.

### Cell isolation and culture

Tissue pieces were washed several times in its own reservoir with ice-cold PBS to empty the gut lumen of its substances. After cleaning, tissues were moved to a Petri dish, cut longitudinally, and the underlying fat in the muscular layer removed. After 4 washes with chelation buffer (2% sorbitol, 1% sucrose, 1% bovine serum albumin fraction V (BSA), 10 μg/ml Gentamicin and 250 ng/ml Amphotericin in Dulbecco’s Phosphate buffered saline), tissues were transferred to a clean Petri dish containing chelation buffer, cut into 2- to 5-mm segments, and fragments transferred to 15 ml-tubes containing fresh chelation buffer supplemented with 1% Collagenase IV/Dispase (Roche, Indianapolis, IN) and 25 μg/ml DNase I (Stem Cell Technology, Cambridge, MA) and vigorously re-suspended up and down with a transfer pipette to mechanically disrupt the tissues. Fragments were incubated in a 37 °C 5% CO_2_ incubator and vigorously dislodged with a transfer pipet every 30 min. After 1.5 h of enzymatic treatment, cells were filtered through a 100 μm filter, and single-cell suspensions cryopreserved in liquid N_2_ until use_._ Infection was performed as described previously with few modifications^8,9,45–48^. Briefly, on the day of infection, isolated cells were thawed, incubated for 2 hs on plain DMEM in a 37^0^C 5% CO_2_ incubator, and exposed or not to wild-type *S*. Typhi strain Ty2 at 1:100 multiplicity of infection (MOI)^8,9^. *S*. Typhi strain Ty2 was obtained through the Center Vaccine Development and Global Health (CVD) cryobank system. After 3 h of incubation, cells were labeled using a panel of 33 metal-labeled monoclonal antibodies (mAbs) (*described below*) (Supplementary Table 1).

### Surface antibodies

Cells were surface stained with anti-human mAbs to CD3 (clone UCHT1), CD4 (clone SK3), CD8 (clone RPA-T8), CD11b (clone ICRF44), CD16 (clone 3G8), CD19 (clone HIB19), CD38 (clone HIT2), CD45 (clone HI30), CD45RO (clone UCHL), CD56 (clone HCD56), CD57 (clone HCD57), CD69 (clone FN50), CD161 (clone HP-3G10), CD163 (clone GHI/61), CCR7 (clone G043H7), EpCAM (CD326, clone 9C4), HLA-DR (clone L243), TCR γδ (clone 11F2)(Fluidigm, Sunnyvale, CA), CD14 (clone 3C10) (Invitrogen, Carlsbad, CA), and TCR Vα7.2 (clone 3C10) (Biolegend, San Diego, CA).

### Intracellular antibodies and metal conjugation

Unconjugated mAbs specific to five histone modifications, one histone variant, and one protein modification (Supplementary Table 1) were labeled with MaxPar X8 labeling kits (Fluidigm) according to the manufacturer’s instructions and used for intracellularly staining. We used mAbs that have been previously validated for use in EpiTOF assays to identify epigenetic and immunological markers at the single-cell level^18,19^.

### Cell staining, data acquisition, and analyses

Mass cytometric assays were performed as previously described with small modifications^49,50^. Briefly, after *S*. Typhi infection, samples were stained for live/dead cell with cisplatin (*194/195 Pt*), followed by two successive 30 min-incubations with human Fc receptor blocking IgG and a metal-labeled antibody cocktail for surface markers to identify 11 major cell subsets (*i.e*., B-, CD3 + T, CD4 + T, CD8 + T, NK, TCR-γδ, Mucosal associated invariant (MAIT) and NKT cells as well as monocytes, macrophages, and epithelial cells). Cells were then fixed and permeabilized, and intracellularly stained with metal-labeled mAbs specific to 6 histone modifications (*i.e*., lysine methylation, acetylation, phosphorylation, citrullination, and ubiquitination, as well as arginine methylation) and 1 histone variant (Supplementary Tables 1 & 2). Finally, samples were stained with an Ir^191^^/193^ DNA intercalator for cell detection by mass cytometry within 48 h of sample acquisition and re-suspended in EQ4 normalization beads (Fluidigm). The acquisition was performed using a Helios mass cytometer (Fluidigm). Eight to 10 × 10^6^ cells were analyzed per volunteer per experiment by mass cytometry. Cell lineages were identified using the manual gating procedure described in Supplementary Fig. 1. Multifunctionality analyses were performed using the FCOM function of WinList software v9.0 (Verity Software House, Topsham, ME) (https://www.vsh.com/products/winlist/index.asp) to determine the proportion of all possible combinations of the 12 measured biomarkers**.** Flow cytometry experiments were performed at the Flow Cytometry and Mass Cytometry Core Facility of the University of Maryland School of Medicine Center for Innovative Biomedical Resources (CIBR), Baltimore, Maryland.

### Statistical analysis

To visualize the variance in the chromatin profiles between and within cell subtypes, we performed a t-Distributed Stochastic Neighbor Embedding (t-SNE)^20^ map followed by the downstream analysis of the data using a self-organizing map (FlowSOM)^21^ via Cytobank (Santa Clara, CA), a cloud-based platform. For t-SNE analysis, we used equal sampling to visualize the differences and relative abundance among the populations across files. The settings for the number of iterations (*i.e*., 3,000), perplexity (*i.e*., 40), and theta (*i.e*., 0.5) were based on the optimal separation of the populations on the tSNE maps. For FlowSOM analysis, hierarchical consensus clustering was used to subsample the events several times to generate a ranked clustering for each subsampling. This step was followed by 10 iterations. The samples were clustered into 225 clusters that were subsequently clustered into 15 meta-clusters based on their similarities in high-dimensional space (*i.e*., considering the relative detection intensities of 12 markers specific to 5 histone modifications, one histone variant, and one protein modification). The resulting clustering was visualized in minimal spanning trees (MST)^51,52^ that connect similar clusters adjacent to each other, using the algorithm proposed by Kamada and Kawai^53^.

To visualize the variance of 28 possible FCOM phenotypes between and within cell subtypes, we performed Principal Component Analysis (PCA). The calculation of principal components was performed by ClustVis web tools^54^. The hierarchical clustering of the heatmap started with calculating all pairwise distances. Objects with the smallest distance were merged in each step. The clustering method defined how to go from the object level to cluster-level when calculating the distance between two clusters. Here, rows were centered, and unit variance scaling was applied to the rows. Rows were clustered using Euclidean distance (square root of the sum of square distances) and average linkage (average distance of all possible pairs) calculated. Columns were clustered using correlation distance (Pearson correlation subtracted from 1) and average linkages calculated. Trees ordering for both rows and columns display the tightest cluster first.

Comparisons between uninfected and *S*. Typhi-infected cultures were carried out by paired Student’s t-tests (Prism software, version 7, GraphPad Software, La Jolla, CA). *P* values < 0.05 were considered significant.

## Supplementary information

Supplementary Information.
