# Author Correction: *Salmonella enterica* serovar Typhi exposure elicits ex vivo cell-type-specific epigenetic changes in human gut cells

**DOI:** 10.1038/s41598-020-79464-y

**Published:** 2021-01-04

**Authors:** Marcelo B. Sztein, Andrea C. Bafford, Rosângela Salerno-Goncalves

**Affiliations:** 1 Center for Vaccine Development and Global Health, Department of Pediatrics, University of Maryland School of Medicine, 685 West Baltimore Street, HSF 2, Room 480, Baltimore, MD 21201 USA; 2 Program in Oncology, University of Maryland Marlene and Stewart Greenebaum Comprehensive Cancer Center, Baltimore, MD 21201 USA; 3 Division of General and Oncologic Surgery, University of Maryland School of Medicine, Baltimore, MD 21201 USA

Correction to: *Scientific Reports* 10.1038/s41598-020-70492-2, Published: 12 August 2020

The original version of this Article contained errors in the spelling of the authors Marcelo B. Sztein and Andrea C. Bafford which were incorrectly given as M. B. Sztein and A. C. Bafford respectively.

Furthermore, in the Author Contributions section the initials of the authors R.S.-G., A.C.B. and M.B.S. were incorrectly given as R.S., A.B. and M.S.

Finally, the Supplementary Information file contained errors in the spelling of the authors Marcelo B. Sztein, Andrea C. Bafford and Rosângela Salerno-Goncalves which were incorrectly given as M. B. Sztein, A. C. Bafford and R. Salerno‐Goncalves respectively.

These errors have now been corrected in the PDF and HTML versions of the Article, and in the accompanying Supplementary Information file.

