# Supplementary material for: Salmonella enterica serovar Typhi exposure elicits ex vivo cell-type-specific epigenetic changes in human gut cells
Source: Sci Rep. 2020 Aug 12;10:13581. doi: 10.1038/s41598-020-70492-2 (PMC7423951; doi:10.1038/s41598-020-70492-2)
Supplement: Supplementary file 1 — Supplementary Information. [file 41598_2020_70492_MOESM1_ESM.pdf]

# *Salmonella enterica* serovar Typhi exposure elicits *ex vivo* cell-type-specific epigenetic changes in human gut cells

M.B. Sztein, A.C. Bafford, and R. Salerno-Goncalves

# Supplementals

**Supplementary table 1.** Antibody panel used to evaluate chromatine changes

| | Atomic Mass: | Markers |
|---|---|---|
| 1 | 89 Y | CD45 |
| 2 | 114 Cd | CD14 |
| 3 | 141 Pr | EpCAM |
| 4 | 142 Nd | γ-H2AX |
| 5 | 143 Nd | HLA-DR |
| 6 | 144 Nd | CD38 |
| 7 | 145 Nd | CD163 |
| 8 | 146 Nd | CD8 |
| 9 | 148 Nd | CD16 |
| 10 | 149 Sm | CD45RO |
| 11 | 150 Nd | H3 |
| 12 | 152 Sm | TCRγδ |
| 13 | 153 Eu | Arg-me2 (asy) |
| 14 | 154 Sm | H3K9me1 |
| 15 | 155Gd | H2BK5ac |
| 16 | 156 Gd | H4K20me3 |
| 17 | 159 Tb | CD161 |
| 18 | 160 Gd | H2BK120ub |
| 19 | 162 Dy | CD69 |
| 20 | 163 Dy | H3K4me3 |
| 21 | 164 Dy | H3K14ac |
| 22 | 165 Ho | TCR Vα7.2 |
| 23 | 166 Er | H4K16ac |
| 24 | 167 Er | CCR7 |
| 25 | 169 Tm | CD19 |
| 26 | 170 Er | CD3 |
| 27 | 171 Yb | H4K20me1 |
| 28 | 172 Yb | CD57 |
| 29 | 173 Yb | PADI4 |
| 30 | 174 Yb | CD4 |
| 31 | 175Lu | Macro-H2A |
| 32 | 176 Yb | CD56 |
| 33 | 209 Bi | CD11b |
| | **Atomic Mass:** | **Other:** |
| 34 | 191/193 Ir | DNA content |
| 35 | 194/195 Pt | Live/dead stain |

**Supplementary table 2.** Epigenetic modifications evaluated in this manuscript

| | Antibody | | |
|---|---|---|---|
| **Chromatine profile** | **Clone** | **Vendor** | **Cat. #** |
| Histones | | | |
| H3 | D1H2 | Cell Signaling Technology | 4499S |
| Histone Variant | | | |
| Macro-H2A | 14G7 | Millipore | MABE61 |
| Post-translational modifications | | | |
| *Lysine Methylation* | | | |
| H4K20me1 | 5E10-D8 | BioLegend | 828001 |
| H4K20me3 | 6F8-D9 | Biolegend | 827701 |
| H3K9me1 | 7E7.H12 | Biolegend | 824201 |
| H3K4me3 | G.532.8 | ThermoFisher | MA5-11199 |
| *Acetylation* | | | |
| H2BK5ac | D5H1S | Cell Signaling Technology | 12799S |
| H3K14ac | D4B9 | Cell Signaling Technology | 7627S |
| H4K16ac | E2B8W | Cell Signaling Technology | 13534S |
| *Phosphorylation* | | | |
| γ-H2AX | 20E3 | Cell Signaling Technology | 9718S |
| *Citrullination* | | | |
| PADI4 | OTI4H5 | OriGene | CF504813 |
| *Ubiquitination* | | | |
| H2BK120ub | D11 | Cell Signaling Technology | 5546S |
| *Arginine Methylation* | | | |
| Arg-me2 (asy) | 13522 | Cell Signaling Technology | 13522S |

**Supplementary Fig. 1**. Gating procedure to identify cell lineage

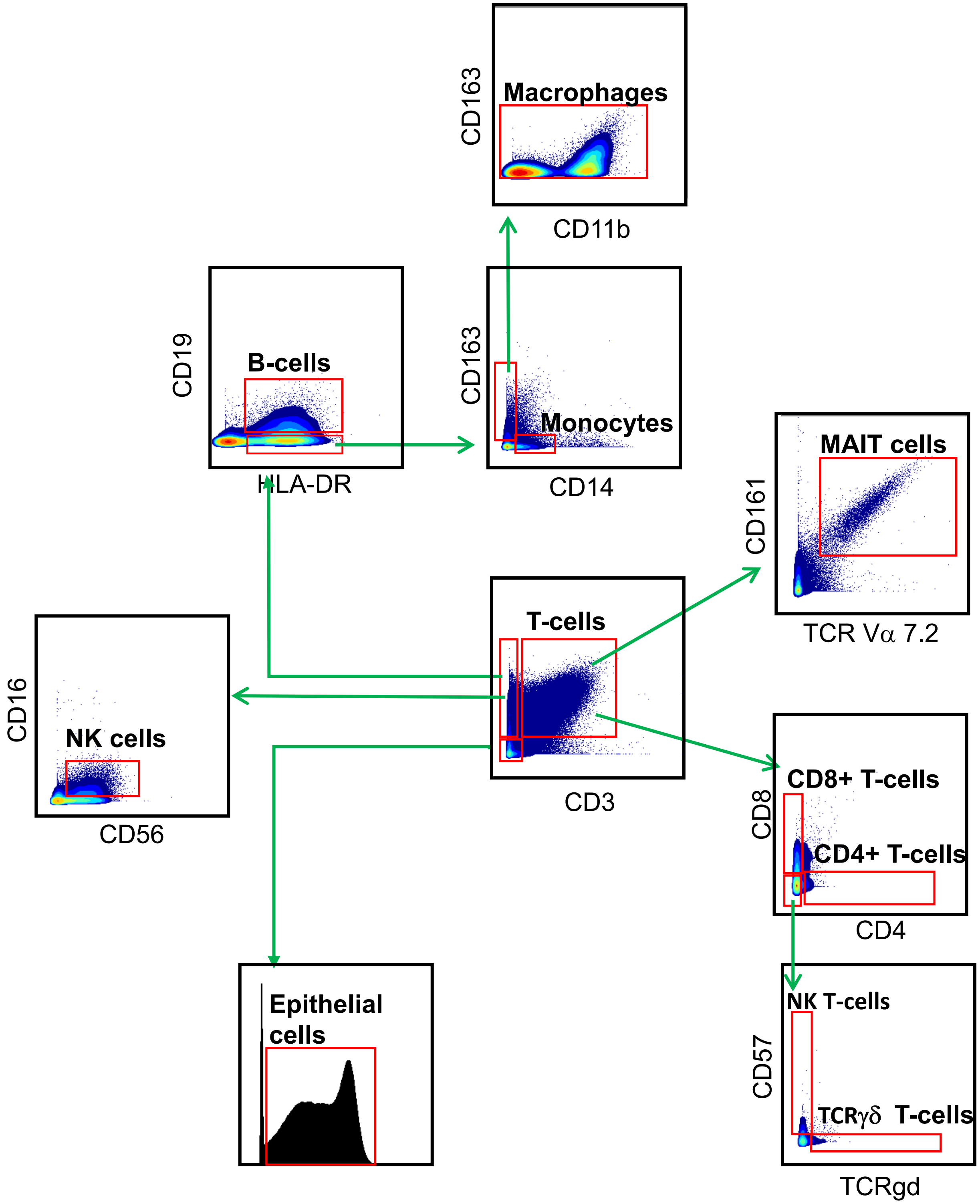

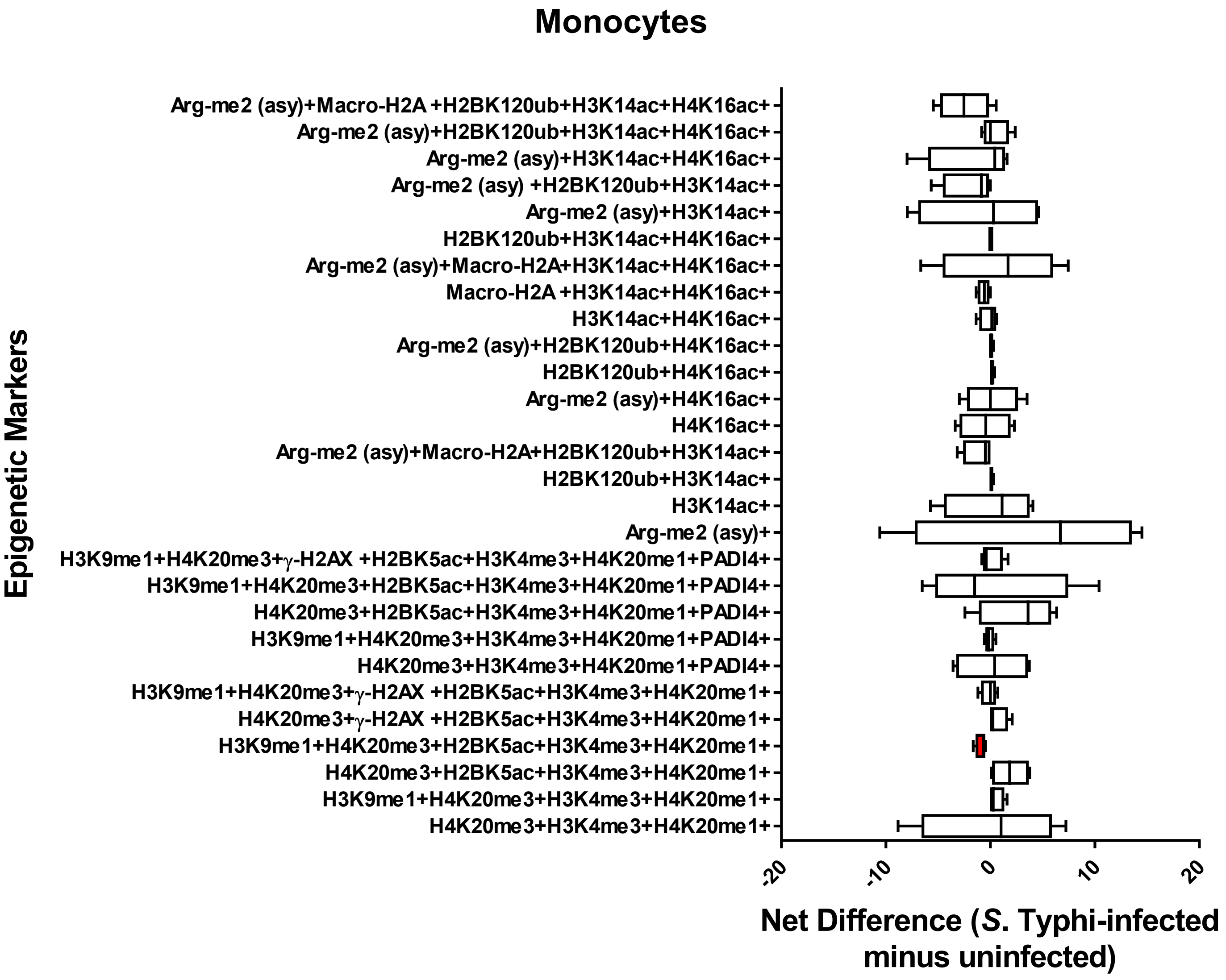


**Supplementary Fig. 2. Chromatin profiles of the epigenetic changes in monocytes induced by *S.* Typhi**. Cells isolated cells from healthy terminal ileum surgical tissues were exposed to *S.* Typhi strain Ty2 (**Ty2**), and cultured as described in **Fig. 1**. Cells cultured with media only were used as controls (**media**). FCOM data of the 28 combinations within the acceptability criteria for changes of the chromatin marks are shown. Bars represent the net difference (*S*. Typhi-infected minus uninfected cultures). Bar graphs extend from the 25$^{th}$ to 75$^{th}$ percentiles; the line in the middle represents the median of the pooled data. The whiskers delineate the smallest to the largest value. These data are representative of three experiments with terminal ileum segments from 4 different donors, one replicate each. *P* values <0.05 were considered significant (red-colored box).

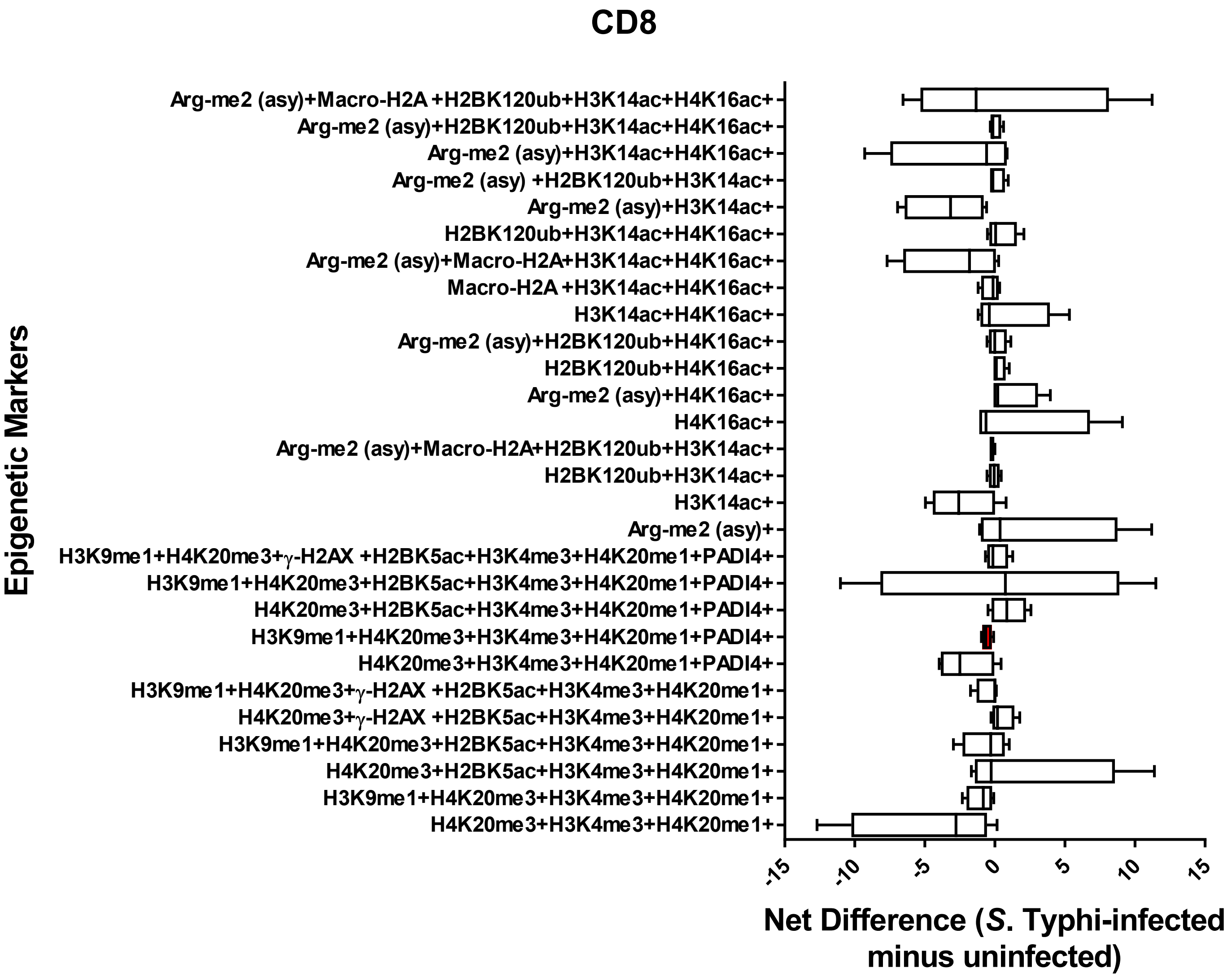


**Supplementary Fig. 3. Chromatin profiles of the epigenetic changes in CD8+ T-cells induced by *S.* Typhi**. Cells isolated cells from healthy terminal ileum surgical tissues were exposed to *S.* Typhi strain Ty2 (**Ty2**), and cultured as described in **Fig. 1**. Cells cultured with media only were used as controls (**media**). FCOM data of the 28 combinations within the acceptability criteria for changes of the chromatin marks are shown. Bars represent the net difference (*S.* Typhi-infected minus uninfected cultures). Bar graphs extend from the 25th to 75th percentiles; the line in the middle represents the median of the pooled data. The whiskers delineate the smallest to the largest value. These data are representative of three experiments with terminal ileum segments from 4 different donors, one replicate each. *P* values <0.05 were considered significant (red-colored box).

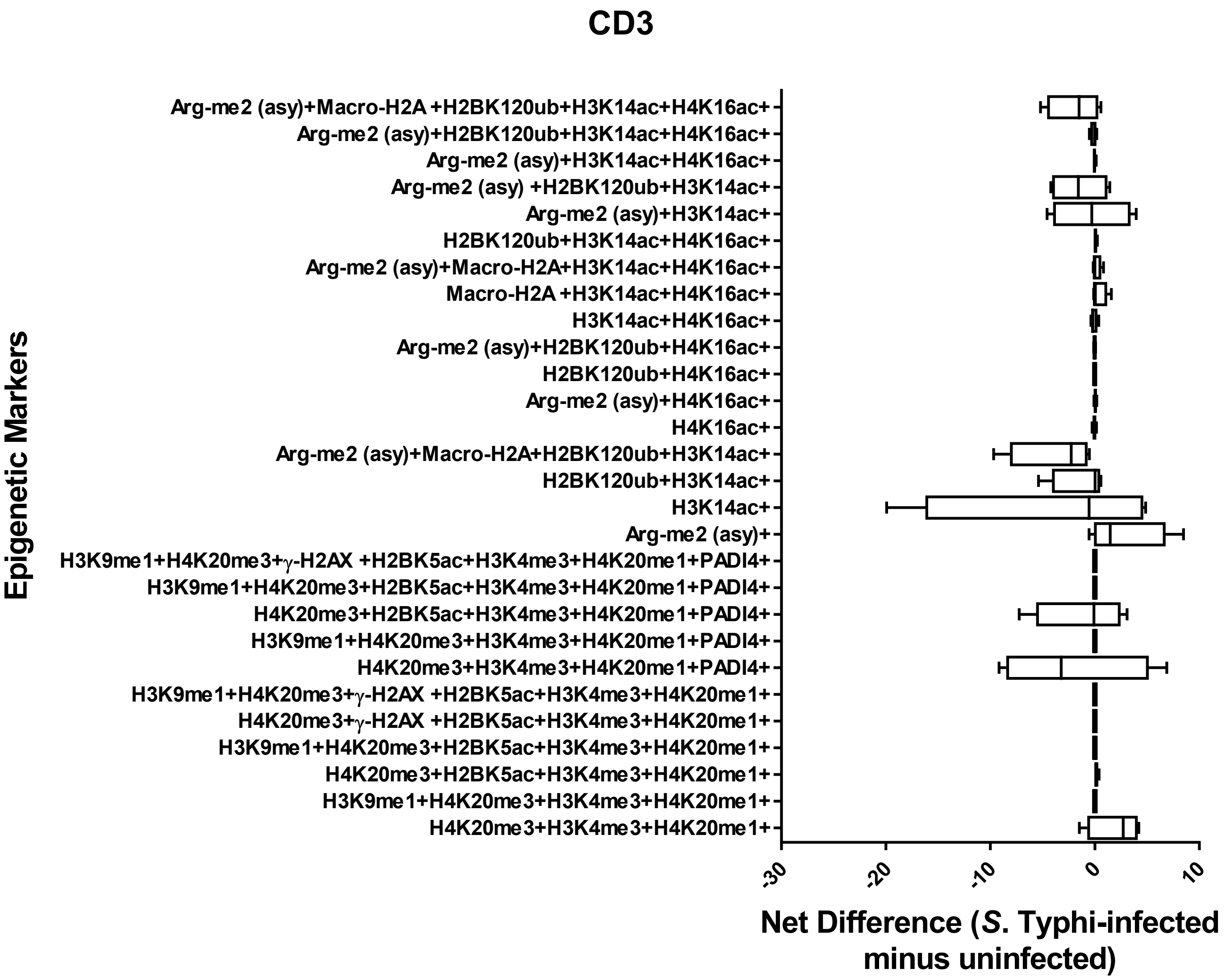


**Supplementary Fig. 4. Chromatin profiles of the epigenetic changes in CD3+ T-cells induced by *S*. Typhi**. Cells isolated cells from healthy terminal ileum surgical tissues were exposed to *S*. Typhi strain Ty2 (**Ty2**), and cultured as described in **Fig. 1**. Cells cultured with media only were used as controls (**media**). FCOM data of the 28 combinations within the acceptability criteria for changes of the chromatin marks are shown. Bars represent the net difference (*S*. Typhi-infected minus uninfected cultures). Bar graphs extend from the 25th to 75th percentiles; the line in the middle represents the median of the pooled data. The whiskers delineate the smallest to the largest value. These data are representative of three experiments with terminal ileum segments from 4 different donors, one replicate each.

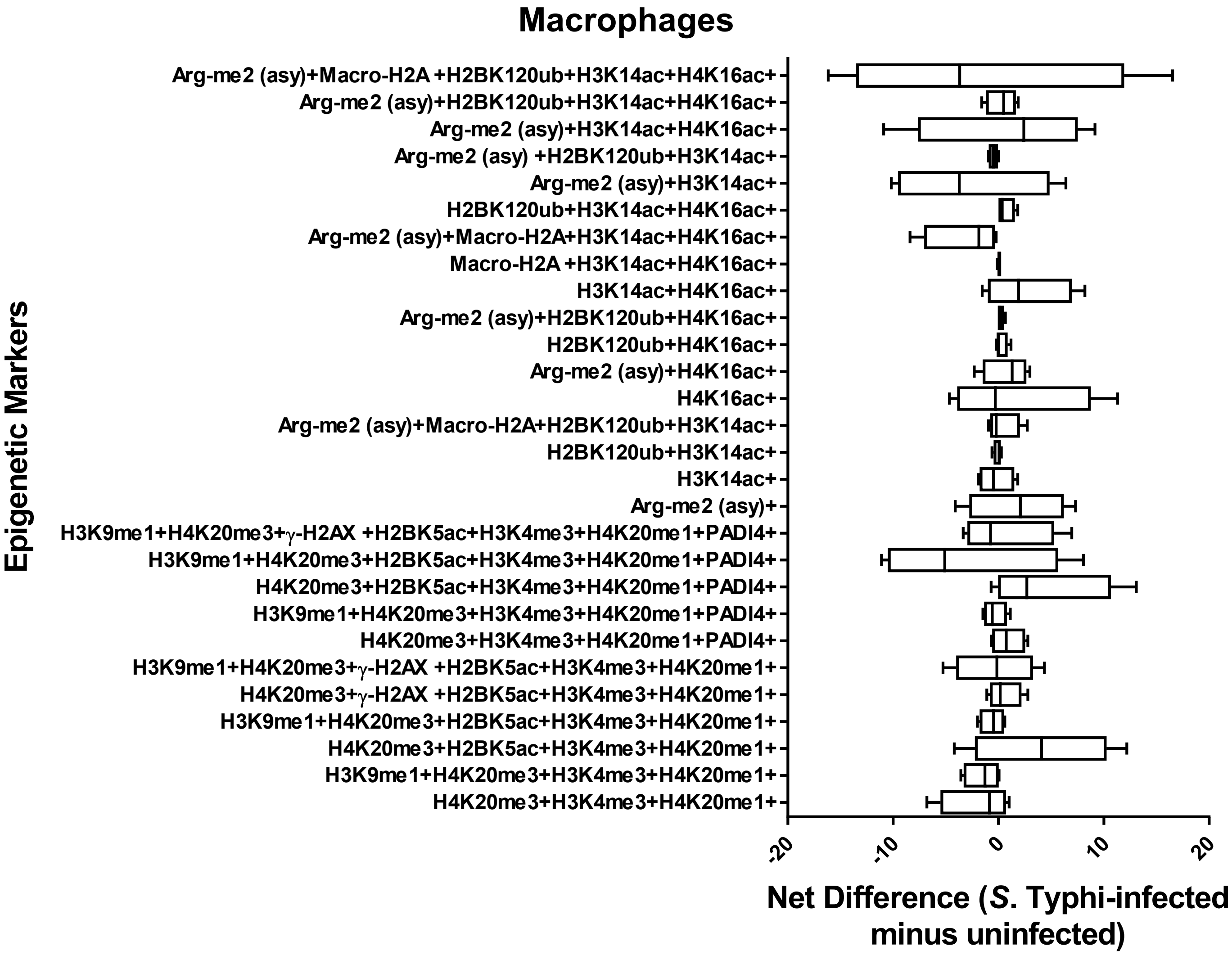


**Supplemental Fig. 5. Chromatin profiles of the epigenetic changes in macrophages induced by *S.* Typhi**. Cells isolated cells from healthy terminal ileum surgical tissues were exposed to *S.* Typhi strain Ty2 (**Ty2**), and cultured as described in **Fig. 1**. Cells cultured with media only were used as controls (**media**). FCOM data of the 28 combinations within the acceptability criteria for changes of the chromatin marks are shown. Bars represent the net difference (*S*. Typhi-infected minus uninfected cultures). Bar graphs extend from the 25$^{th}$ to 75$^{th}$ percentiles; the line in the middle represents the median of the pooled data. The whiskers delineate the smallest to the largest value. These data are representative of three experiments with terminal ileum segments from 4 different donors, one replicate each.

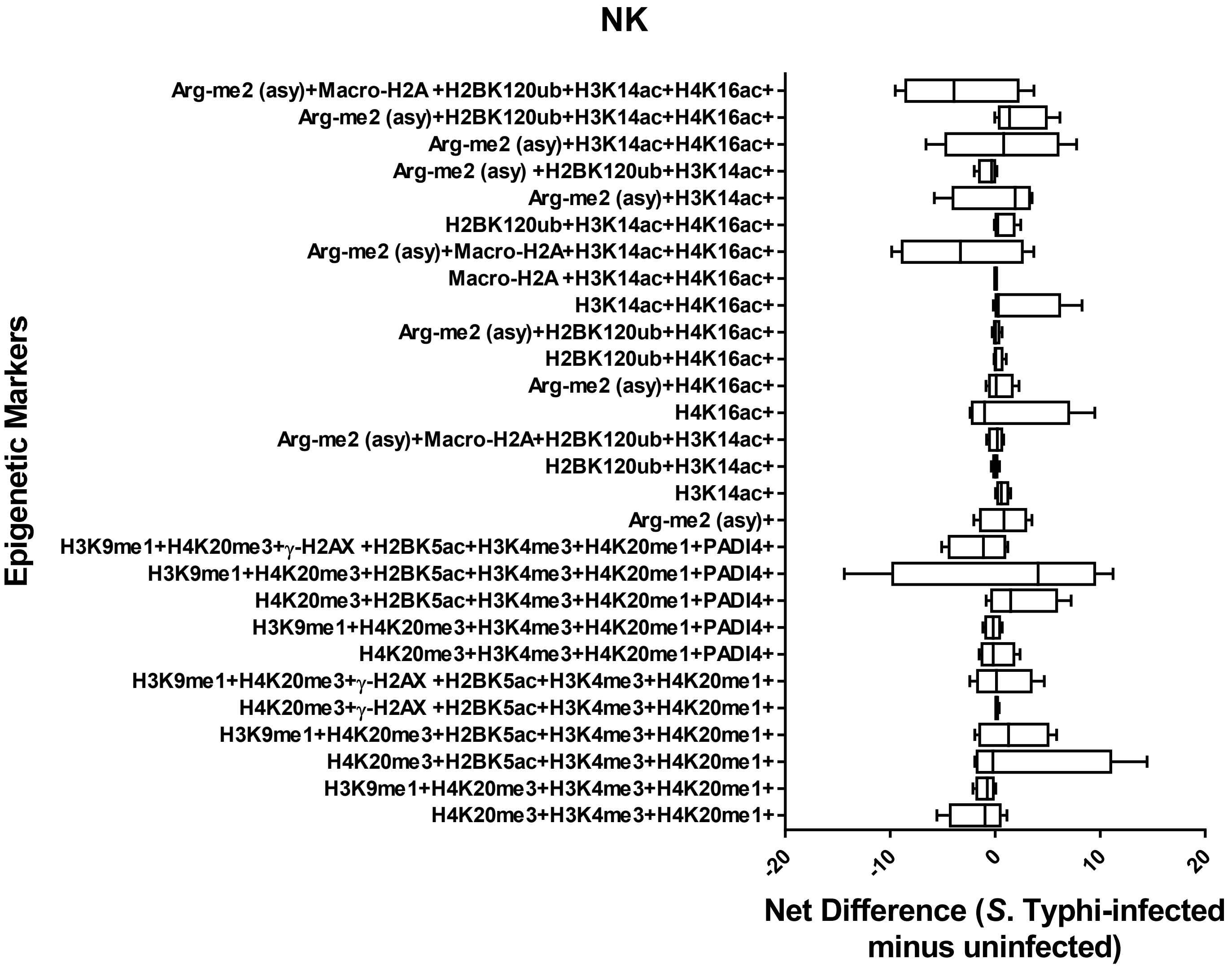


**Supplemental Fig. 6. Chromatin profiles of the epigenetic changes in NK cells induced by *S.* Typhi**. Cells isolated cells from healthy terminal ileum surgical tissues were exposed to *S*. Typhi strain Ty2 (**Ty2**), and cultured as described in **Fig. 1**. Cells cultured with media only were used as controls (**media**). FCOM data of the 28 combinations within the acceptability criteria for changes of the chromatin marks are shown. Bars represent the net difference (*S*. Typhi-infected minus uninfected cultures). Bar graphs extend from the 25th to 75th percentiles; the line in the middle represents the median of the pooled data. The whiskers delineate the smallest to the largest value. These data are representative of three experiments with terminal ileum segments from 4 different donors, one replicate each.

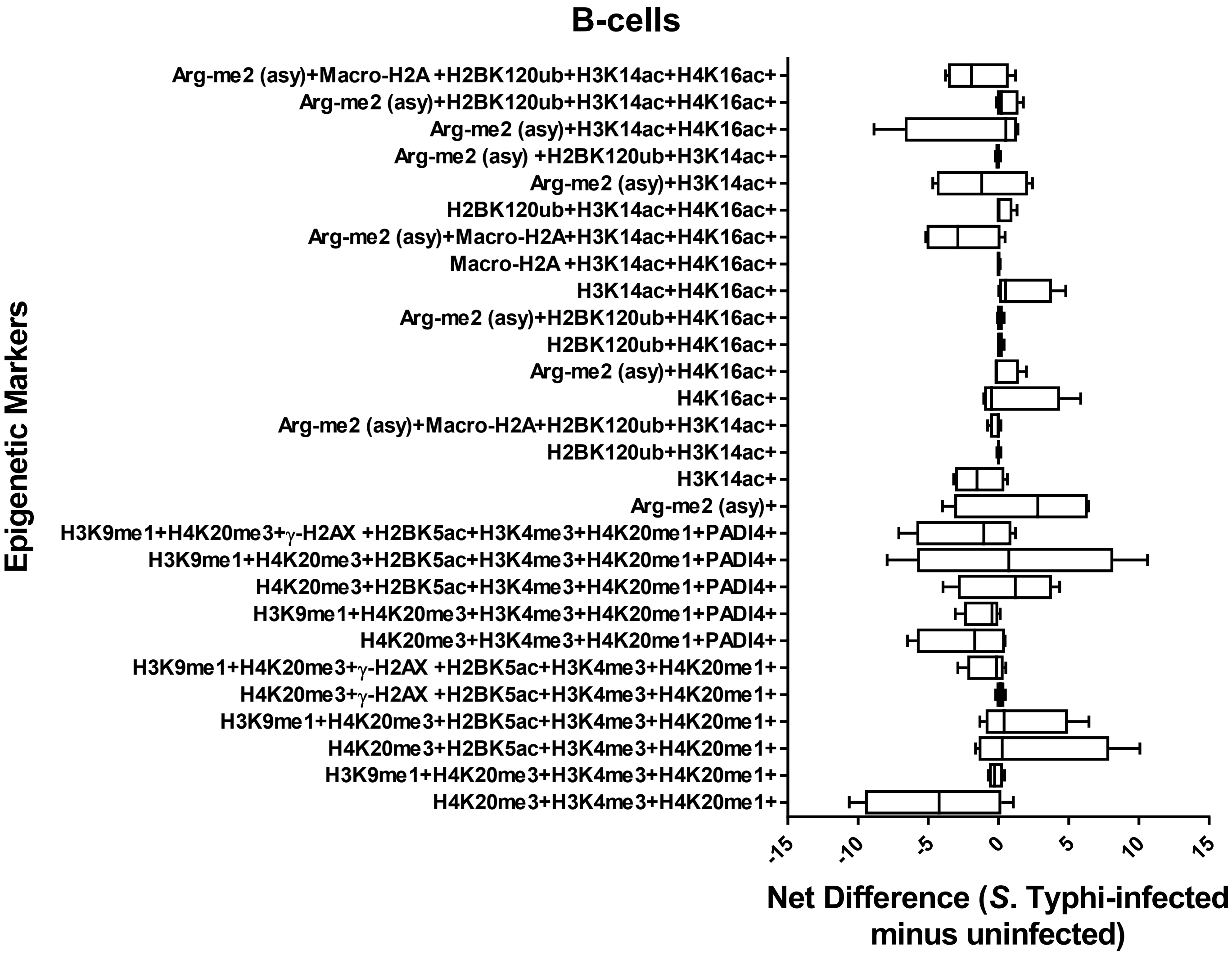


**Supplemental Fig. 7. Chromatin profiles of the epigenetic changes in B-cells induced by *S.* Typhi**. Cells isolated cells from healthy terminal ileum surgical tissues were exposed to *S*. Typhi strain Ty2 (**Ty2**), and cultured as described in **Fig. 1**. Cells cultured with media only were used as controls (**media**). FCOM data of the 28 combinations within the acceptability criteria for changes of the chromatin marks are shown. Bars represent the net difference (*S*. Typhi-infected minus uninfected cultures). Bar graphs extend from the 25$^{th}$ to 75$^{th}$ percentiles; the line in the middle represents the median of the pooled data. The whiskers delineate the smallest to the largest value. These data are representative of three experiments with terminal ileum segments from 4 different donors, one replicate each, one replicate each.

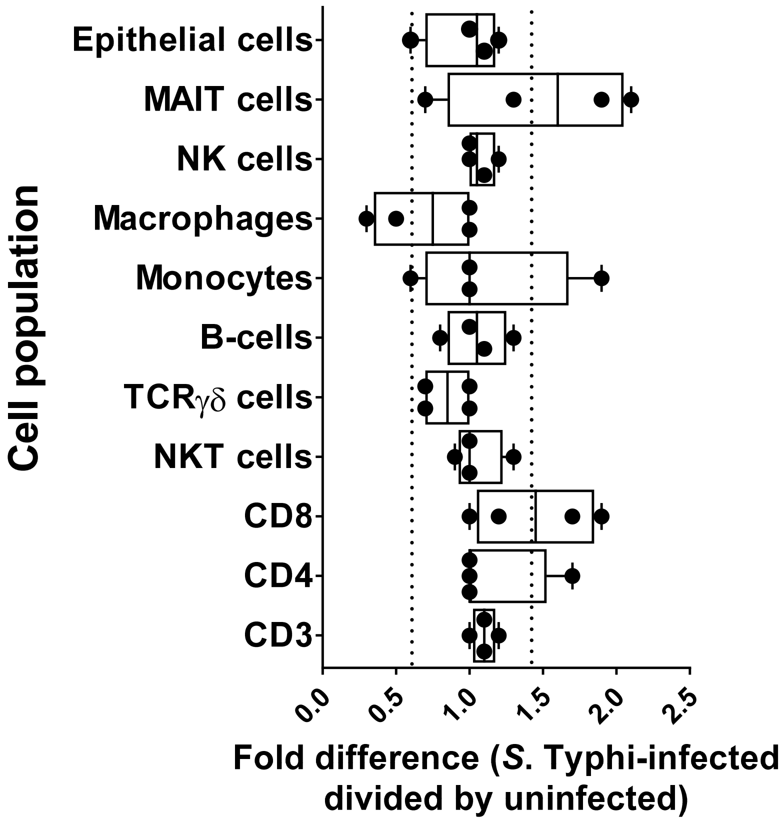


**Supplemental Fig. 8. Chromatin profile of H3 histone changes in 11 cell subsets induced by *S*. Typhi**. Cells isolated from healthy terminal ileum surgical tissues were exposed to *S*. Typhi strain Ty2 (**Ty2**), and cultured as described in **Fig. 1**. Cells cultured with media only were used as controls (**media**). Bars represent the fold difference (*S*. Typhi-infected divided by uninfected cultures). Bar graphs extend from the 25$^{th}$ to 75$^{th}$ percentiles; the line in the middle represents the median of the pooled data. The whiskers delineate the smallest to the largest value. These data are representative of three experiments with terminal ileum segments from 4 different donors, one replicate each. The doted lines express the data distribution within 95% confidence (± 2SD).

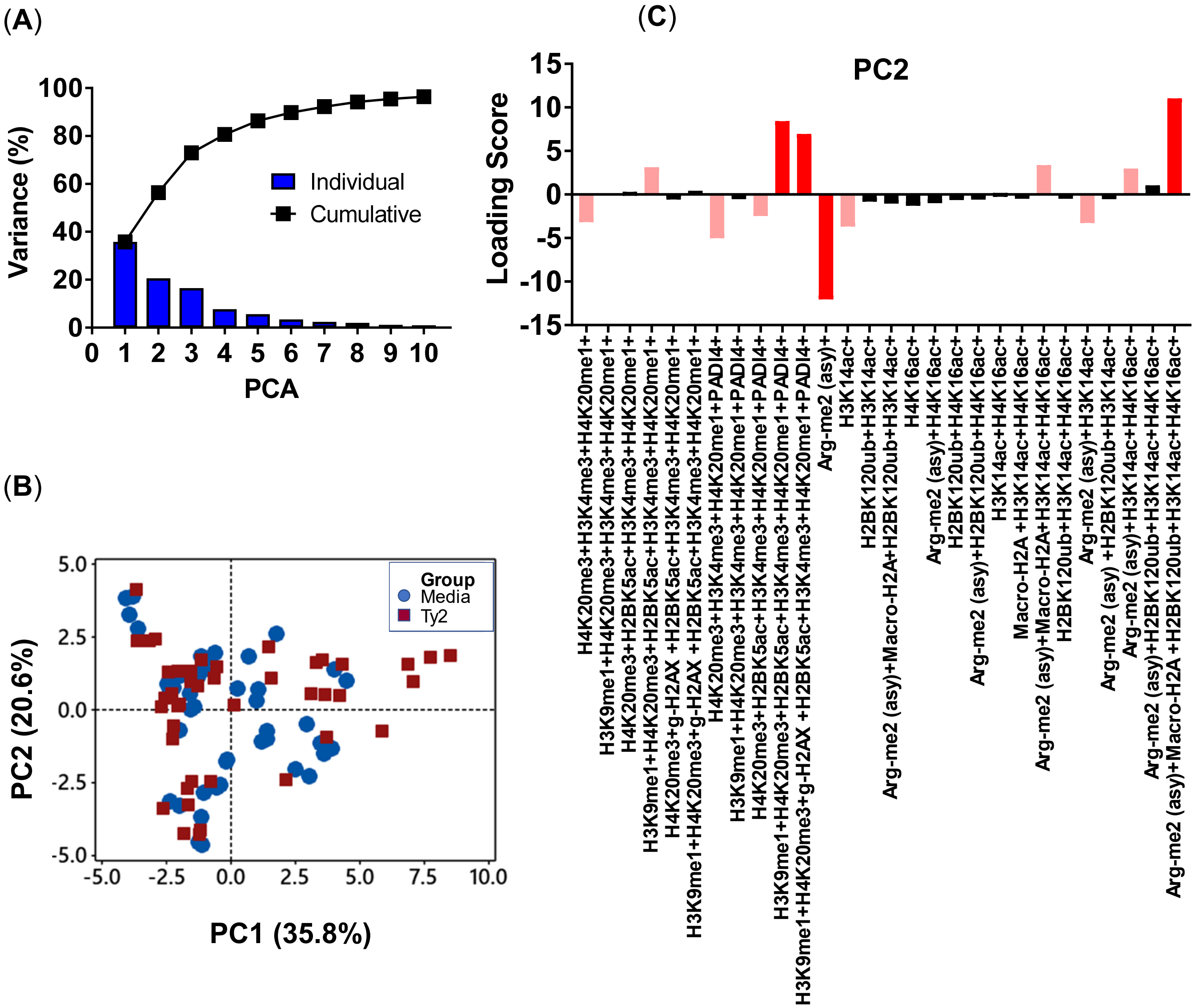


**Supplemental Fig. 9**. **Principal Component Analysis variances**. (**A**) The percent variation are plotted for each component (bars) and cumulatively (line).(**B**) Ability of PCA to separate FCOM data between media and Ty2 conditions. Each dot represents a single experimental condition, where each PC summarizes the variance of 308 data points for each of the 4 volunteers. (**C**) The principal component analysis parameter loadings (weighting coefficients) for the second component (PC2) is plotted. Colored bars represent the phenotypes with high (●) and moderate (●) variances in PC2.

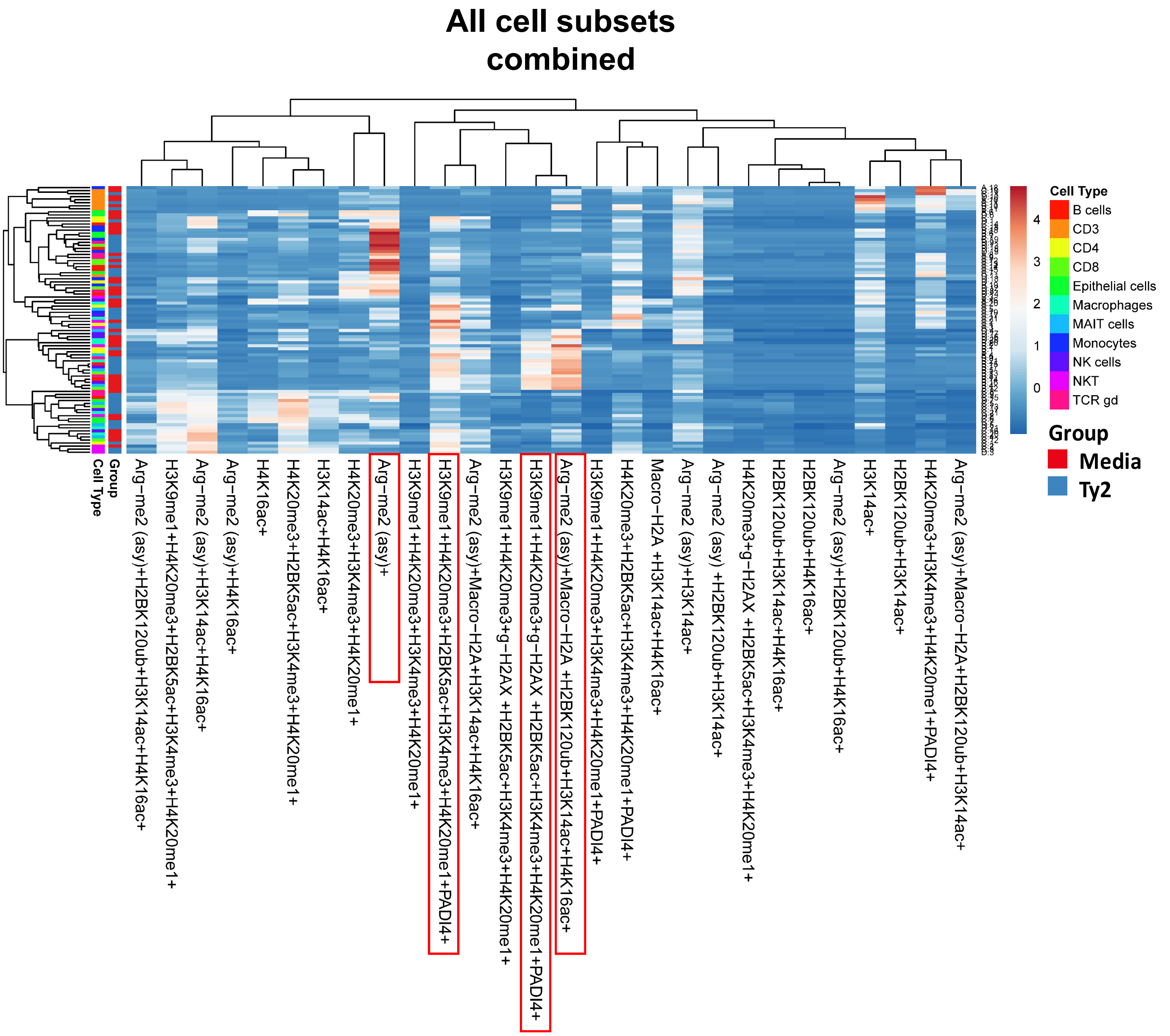


**Supplemental Fig. 10. Hierarchical clustering of all cell subsets using principal component analysis**. Cells isolated from healthy terminal ileum surgical tissues were exposed to *S*. Typhi strain Ty2 (**Ty2**), and cultured as described in **Fig. 1**. Cells cultured with media only were used as controls (**media**). FCOM data of the 28 combinations (within the acceptability criteria for changes of chromatin marks) were used to perform an unsupervised principal component analysis (PCA). PCA compared sample changes before and after exposure to Ty2. PCA was performed using the ClustVis web tool. Rows were centered and unit variance scaling applied to rows. Rows were clustered using Euclidean distance and average linkage. Columns were clustered using correlation distance and average linkage. Trees ordering for both rows and columns display the tightest cluster first. Red boxes represent the phenotypes with high variances in PC2. These data are representative of three experiments with terminal ileum segments from 4 different donors, one replicate each.

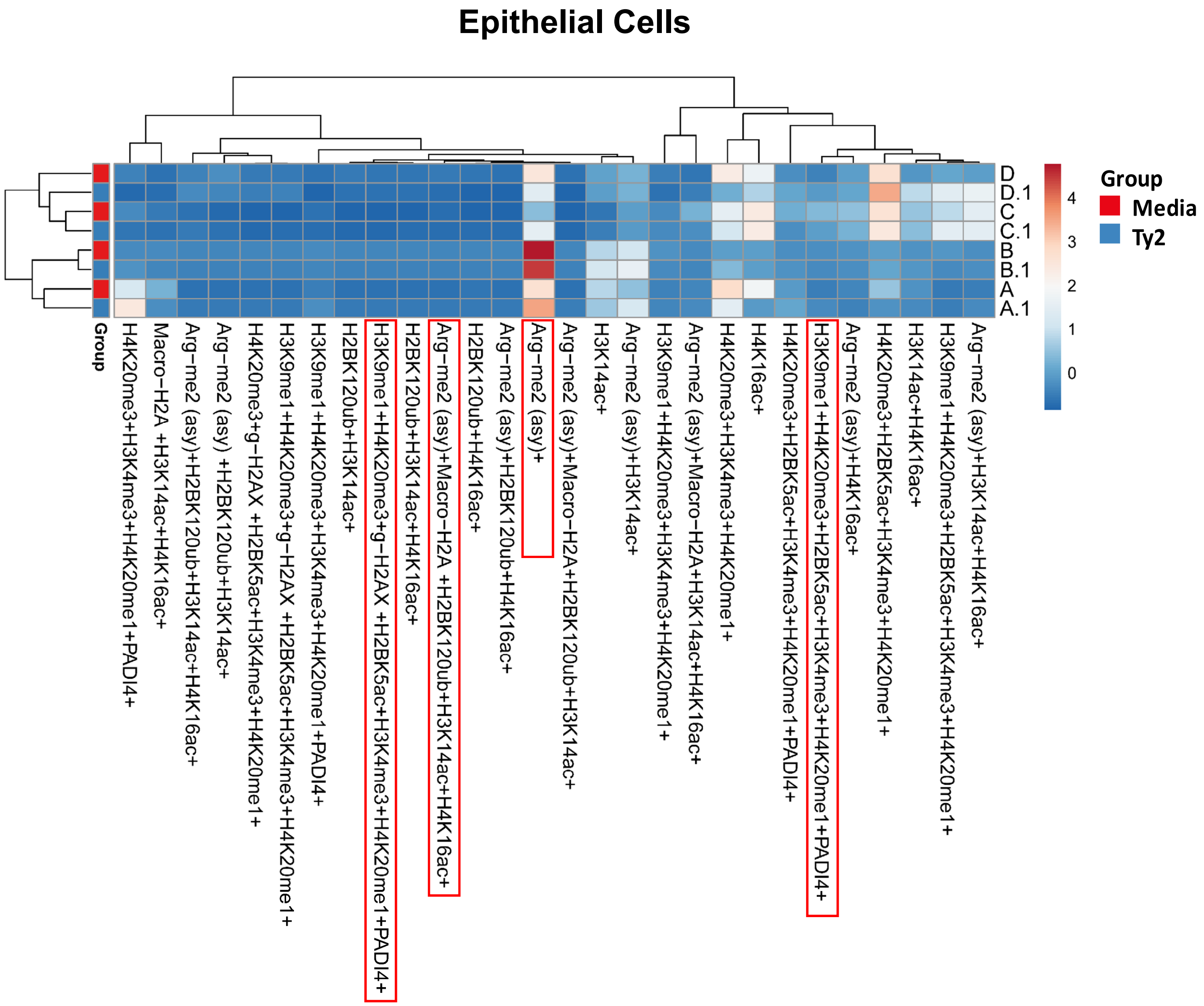


**Supplemental Fig. 11. Hierarchical clustering of epithelial cell population using Principal component analysis**. Cells isolated from healthy terminal ileum surgical tissues were exposed to *S*. Typhi strain Ty2 (**Ty2**), and cultured as described in **Fig. 1**. Cells cultured with media only were used as controls (**media**). FCOM data of the 28 combinations (within the acceptability criteria for changes of chromatin marks) were used to perform an unsupervised principal component analysis (PCA). PCA compared sample changes before and after exposure to Ty2. PCA was performed using the ClustVis web tool. Rows were centered and unit variance scaling applied to rows. Rows were clustered using Euclidean distance and average linkage. Columns were clustered using correlation distance and average linkage. Trees ordering for both rows and columns display the tightest cluster first. Red boxes represent the phenotypes with high variances in PC2. These data are representative of three experiments with terminal ileum segments from 4 different donors, one replicate each.

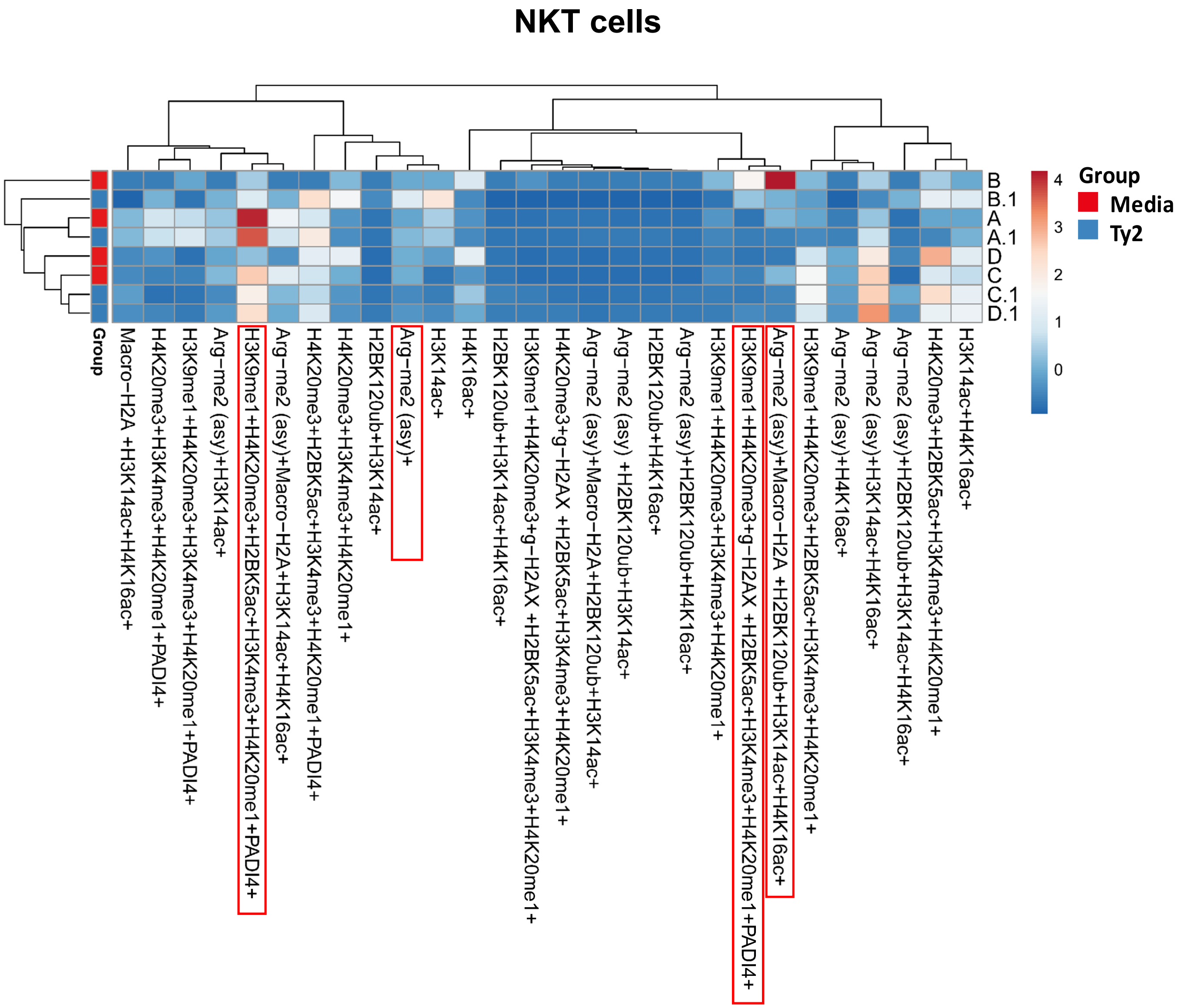


**Supplemental Fig. 12. Hierarchical clustering of NKT cell population using Principal component analysis**. Cells isolated from healthy terminal ileum surgical tissues were exposed to *S*. Typhi strain Ty2 (**Ty2**), and cultured as described in **Fig. 1**. Cells cultured with media only were used as controls (**media**). FCOM data of the 28 combinations (within the acceptability criteria for changes of chromatin marks) were used to perform an unsupervised principal component analysis (PCA). PCA compared sample changes before and after exposure to Ty2. PCA was performed using the ClustVis web tool. Rows were centered and unit variance scaling applied to rows. Rows were clustered using Euclidean distance and average linkage. Columns were clustered using correlation distance and average linkage. Trees ordering for both rows and columns display the tightest cluster first. Red boxes represent the phenotypes with high variances in PC2. These data are representative of three experiments with terminal ileum segments from 4 different donors, one replicate each.

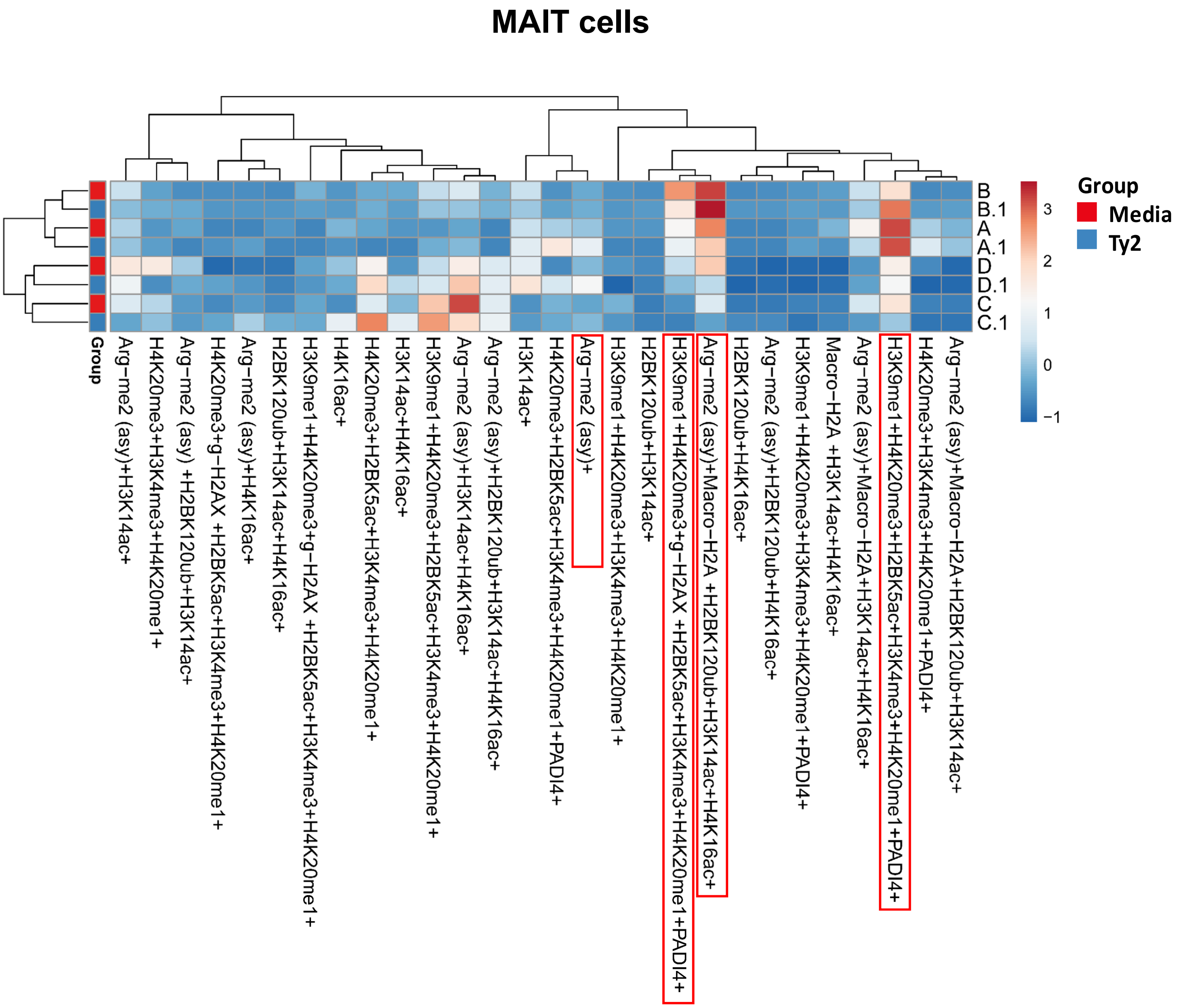


**Supplemental Fig. 13. Hierarchical clustering of MAIT cell population using Principal component analysis**. Cells isolated from healthy terminal ileum surgical tissues were exposed to *S*. Typhi strain Ty2 (**Ty2**), and cultured as described in **Fig. 1**. Cells cultured with media only were used as controls (**media**). FCOM data of the 28 combinations (within the acceptability criteria for changes of chromatin marks) were used to perform an unsupervised principal component analysis (PCA). PCA compared sample changes before and after exposure to Ty2. PCA was performed using the ClustVis web tool. Rows were centered and unit variance scaling applied to rows. Rows were clustered using Euclidean distance and average linkage. Columns were clustered using correlation distance and average linkage. Trees ordering for both rows and columns display the tightest cluster first. Red boxes represent the phenotypes with high variances in PC2. These data are representative of three experiments with terminal ileum segments from 4 different donors, one replicate each.

## TCR-γδ cells

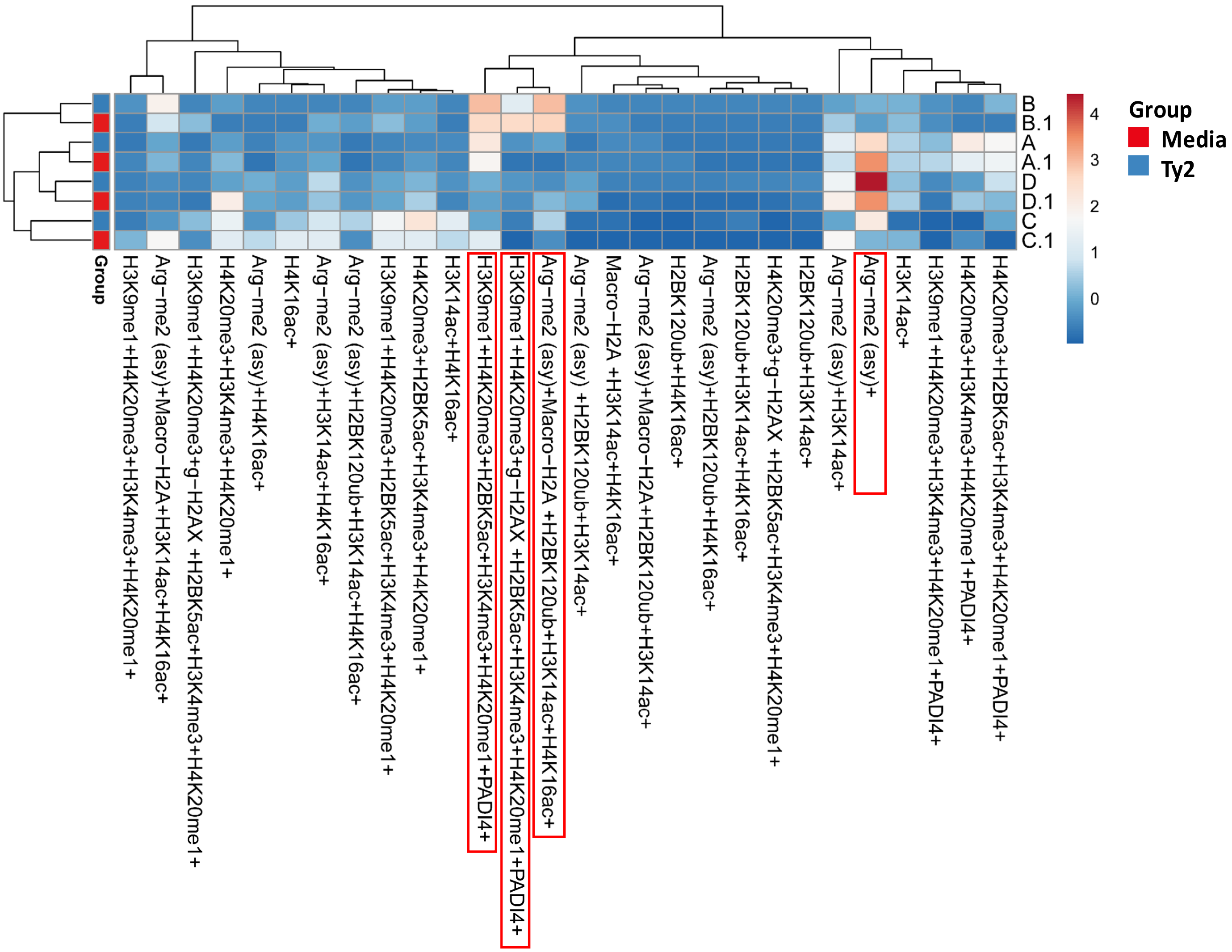


**Supplemental Fig. 14. Hierarchical clustering of TCR-γδ cell population using Principal component analysis**. Cells isolated from healthy terminal ileum surgical tissues were exposed to *S*. Typhi strain Ty2 (**Ty2**), and cultured as described in **Fig. 1**. Cells cultured with media only were used as controls (**media**). FCOM data of the 28 combinations (within the acceptability criteria for changes of chromatin marks) were used to perform an unsupervised principal component analysis (PCA). PCA compared sample changes before and after exposure to Ty2. PCA was performed using the ClustVis web tool. Rows were centered and unit variance scaling applied to rows. Rows were clustered using Euclidean distance and average linkage. Columns were clustered using correlation distance and average linkage. Trees ordering for both rows and columns display the tightest cluster first. Red boxes represent the phenotypes with high variances in PC2. These data are representative of three experiments with terminal ileum segments from 4 different donors, one replicate each.

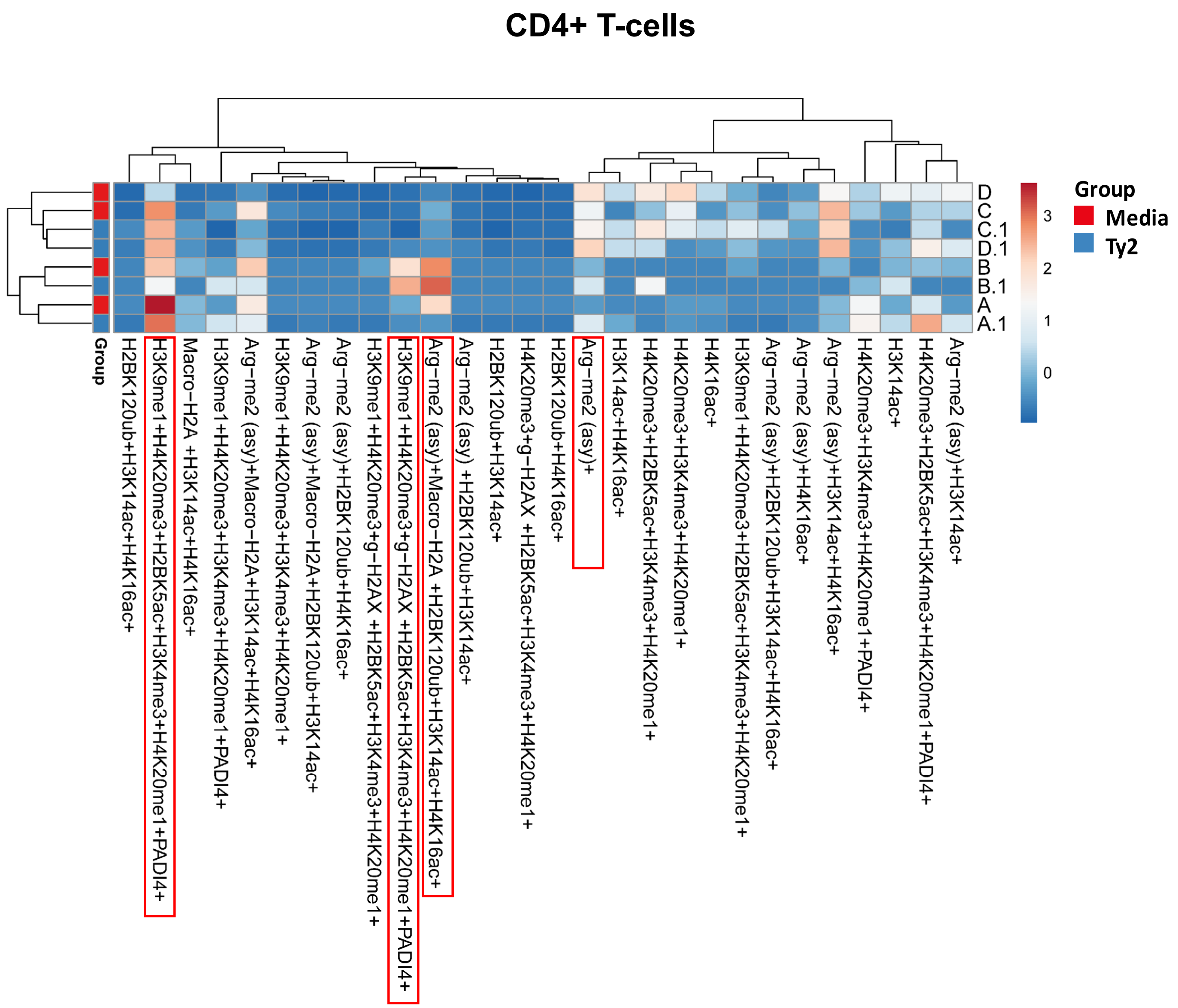


**Supplemental Fig. 15. Hierarchical clustering of CD4+ T-cell population using Principal component analysis**. Cells isolated from healthy terminal ileum surgical tissues were exposed to *S*. Typhi strain Ty2 (**Ty2**), and cultured as described in **Fig. 1**. Cells cultured with media only were used as controls (**media**). FCOM data of the 28 combinations (within the acceptability criteria for changes of chromatin marks) were used to perform an unsupervised principal component analysis (PCA). PCA compared sample changes before and after exposure to Ty2. PCA was performed using the ClustVis web tool. Rows were centered and unit variance scaling applied to rows. Rows were clustered using Euclidean distance and average linkage. Columns were clustered using correlation distance and average linkage. Trees ordering for both rows and columns display the tightest cluster first. Red boxes represent the phenotypes with high variances in PC2. These data are representative of three experiments with terminal ileum segments from 4 different donors, one replicate each.
